# Pharmacological Switching in Minor Phytocannabinoids: A Mechanistic Framework for Compound–Indication Matching and Precision Neurotherapeutic Development

**DOI:** 10.3390/ph19081226

**Published:** 2026-08-04

**Authors:** Amina M. Bagher

**Affiliations:** Department of Pharmacology and Toxicology, Faculty of Pharmacy, King Abdulaziz University, Jeddah 21589, Saudi Arabia; abagher@kau.edu.sa; Tel.: +966-547187973

**Keywords:** cannabidivarin, tetrahydrocannabivarin, cannabigerol, neuroinflammation, 5-HT_1_A receptor, PPARγ, allosteric modulation, biomarker-guided clinical trials

## Abstract

**Background**: Minor phytocannabinoids engage CB_1_ and CB_2_ receptors, as well as broader receptors and ion-channel targets. However, fragmented evidence limits translation into central nervous system (CNS) therapeutics. This review introduces pharmacological switching, defined as a dose-, state-, or model-dependent shift in the dominant determinant of a compound’s net effect that reverses the direction, rather than merely the magnitude, of that effect on a defined functional endpoint, as a framework for compound–indication matching. Unlike biased agonism, which redistributes downstream coupling without reversing direction, switching requires directional reversal between or within receptor systems. **Methods**: This narrative review integrates receptor pharmacology, preclinical CNS disease-model data, early-phase clinical evidence, and pharmacokinetics identified through a structured literature search. **Results**: Three mechanistic axes recurred: PPARγ/CB_2_/Nrf2-linked neuroinflammatory modulation; TRP/GABA_A_/5-HT_1_A-linked control of neuronal excitability; and low-efficacy CB_1_ antagonism with 5-HT_1_A potentiation. Their expression was context-dependent: tetrahydrocannabivarin is reported to shift from CB_1_ antagonism toward partial agonism as receptor occupancy rises, cannabidiolic acid shows stress-conditional anxiolytic and antiemetic activity, and cannabigerolic acid displays seizure-model-dependent bidirectionality. No instance yet meets all five defining criteria; each is graded provisional or proposed pending systematic testing. Human evidence remains limited: inconclusive cannabidivarin efficacy in epilepsy and neuropathic pain, proof-of-mechanism neuroimaging in autism, and early tetrahydrocannabivarin safety data. **Conclusions**: Minor phytocannabinoids are pharmacologically diverse but clinically underdeveloped. A tiered, biomarker-guided framework is proposed to match compound–indication pairs to the doses, formulations, and subgroups most likely to express their therapeutic pharmacology.

## 1. Introduction

*Cannabis sativa* L. produces a chemically diverse group of more than 150 meroterpenoid secondary metabolites known as phytocannabinoids [1,2]. For more than half a century, research and regulatory attention have centered on just two: Δ^9^-tetrahydrocannabinol (Δ^9^-THC) and cannabidiol (CBD). The remainder are conventionally grouped as “minor cannabinoids,” a label that reflects historical neglect rather than natural scarcity [2,3]. This class encompasses neutral cannabinoids such as cannabinol (CBN), cannabichromene (CBC), and cannabigerol (CBG); their carboxylated biosynthetic precursors cannabidiolic acid (CBDA), cannabigerolic acid (CBGA), and tetrahydrocannabinolic acid (Δ^9^-THCA); and the propyl-sidechain varinoids cannabidivarin (CBDV) and Δ^9^-tetrahydrocannabivarin (Δ^9^-THCV) [4,5].

Δ^9^-THC and CBD have yielded two marketed neurotherapeutics: Sativex^®^ (nabiximols; Δ^9^-THC:CBD 1:1) and Epidiolex^®^ (purified CBD). Both, however, carry substantive limitations. Δ^9^-THC is constrained by dose-limiting psychotropic effects (euphoria, anxiety, cognitive impairment) driven by central cannabinoid receptor type 1 (CB_1_) agonism [5,6]. CBD, although non-intoxicating, requires high doses (5–25 mg·kg^−1^·day^−1^), shows erratic oral bioavailability [7], inhibits hepatic cytochrome P450 (CYP) enzymes CYP3A4 and CYP2C19, and generates hepatotoxicity signals at clinically relevant exposures [8,9]. These shortcomings gain urgency in the context of neurodegenerative diseases, where current pharmacotherapy for Huntington’s disease (HD), Parkinson’s disease (PD), and Alzheimer’s disease remains purely symptomatic and fails to arrest the underlying neuronal loss [10,11]. The increasingly restrictive regulatory posture toward intoxicating cannabinoids in the United States and European Union further favors non-intoxicating compounds lacking full CB_1_ agonist efficacy [12,13,14]. Whether minor phytocannabinoids can open mechanistically distinct avenues for central nervous system (CNS) intervention is therefore of increasing translational relevance.

The chief obstacle to answering this question is interpretive fragmentation: minor cannabinoids have been examined across heterogeneous models, targets, and dosing protocols, yielding a literature that resists coherent synthesis. Promising preclinical signals have, in several instances, failed in clinical testing, often because the originating studies lacked antagonist controls for mechanistic assignment or pharmacokinetic confirmation of target-site exposure [15,16,17]. The field, therefore, requires a mechanistic framework that maps target engagement to functional outcomes. Such a framework must be rooted in the endocannabinoid system, comprising the CB_1_ and cannabinoid receptor type 2 (CB_2_) receptors, the endogenous ligands anandamide (AEA) and 2-arachidonoylglycerol (2-AG), and the catabolic enzymes fatty acid amide hydrolase (FAAH) and monoacylglycerol lipase (MAGL) [18,19,20]. Crucially, it must also accommodate the substantial engagement of these compounds at transient receptor potential (TRP) channels, serotonergic 5-hydroxytryptamine receptor type 1A (5-HT_1_A) receptors, and peroxisome proliferator-activated receptors (PPARs). Determining when this multi-target engagement produces therapeutic synergy and when it instead disperses or reverses the intended signal is the central analytical problem this review addresses.

Several prior reviews have addressed overlapping territory. Walsh et al. [5] mapped phytocannabinoid biosynthesis and molecular pharmacology. Stone et al. [4] systematically evaluated neuroprotective mechanisms. Basavarajappa and Subbanna [21] presented a narrative synthesis of CBD-related minor phytocannabinoids (CBG, CBDV, Δ^9^-THCV) for neurological disorders, framing their candidacy based on structural homology to CBD rather than on independent receptor pharmacology. Cammà et al. [3] conducted a systematic review of psychiatric applications. Hakami and Alshehri [22] reviewed clinical trial evidence for cannabinoids in neurological conditions in this journal, focusing on Δ^9^-THC and CBD, but did not systematically profile individual minor phytocannabinoids at the receptor level or integrate receptor pharmacology with translational barriers.

No prior review, however, has used pharmacological switching as an organizing framework to integrate receptor pharmacology, preclinical disease modeling, clinical outcomes, pharmacokinetics, and biomarker-guided prioritization across the minor cannabinoid class. We critically evaluate eight minor phytocannabinoids (CBG, CBC, CBN, CBDA, CBGA, Δ^9^-THCA, CBDV, and Δ^9^-THCV) across four objectives: (i) mapping molecular pharmacology at CB_1_/CB_2_, TRP, 5-HT_1_A, and PPAR targets; (ii) evaluating preclinical evidence in models of epilepsy, neurodegeneration, neuropsychiatric illness, pain, and substance use; (iii) appraising the available clinical dataset; and (iv) identifying the translational bottlenecks that currently separate benchside promise from bedside application. The scope is deliberately restricted to non-intoxicating or minimally psychoactive compounds. Semi-synthetic derivatives are included when they illuminate the parent phytocannabinoid’s mechanism; VCE-003.2 receives fuller treatment given its consistent neuroprotective evidence across multiple disease models. Beyond conventional evidence synthesis, this review formalizes the concept of pharmacological switching, annotates findings by replication status where the evidence permits, and proposes a biomarker-guided translational roadmap.

## 2. Methods and Search Strategy

**Figure 1:**
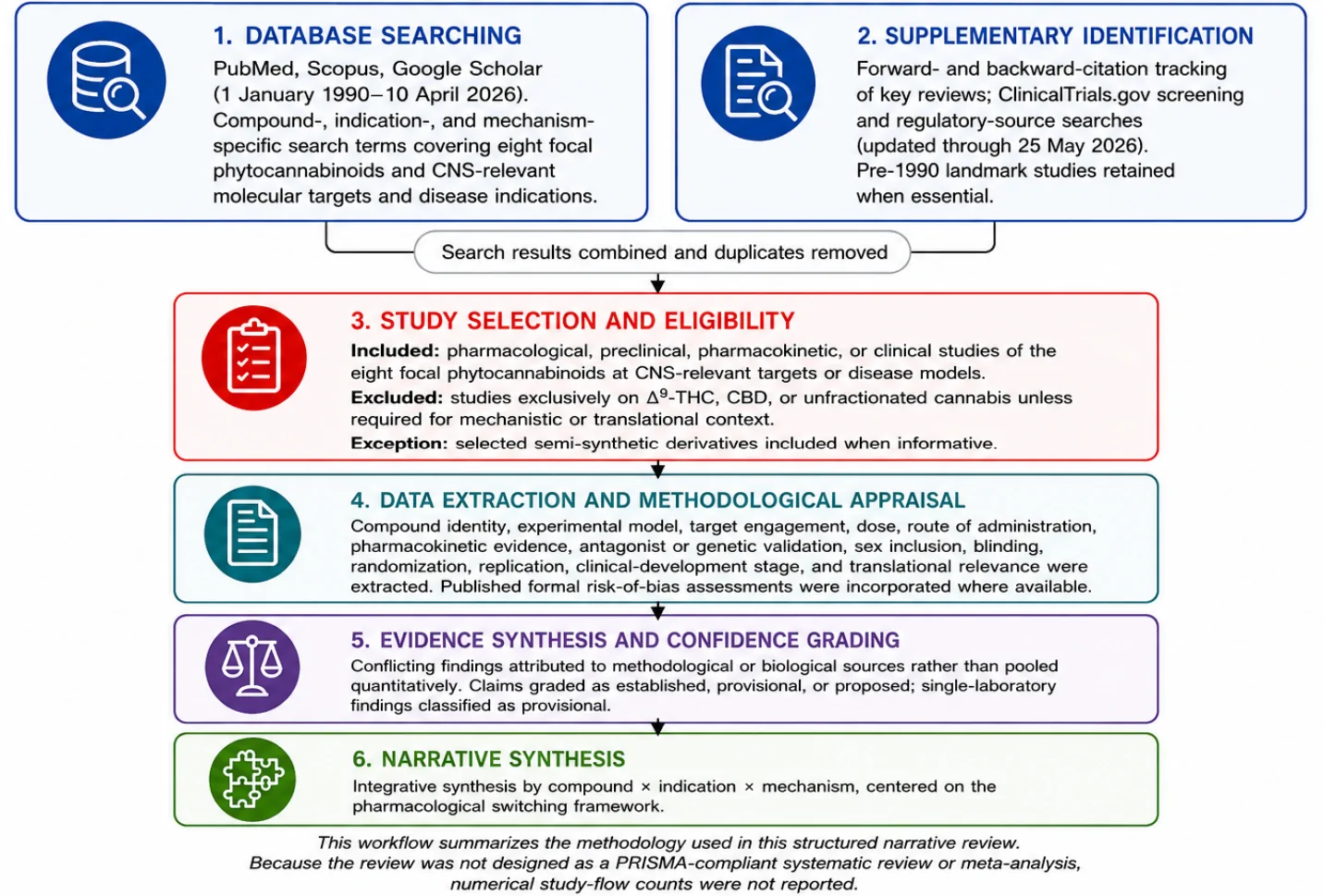
Workflow for literature search, study selection, and narrative evidence synthesis. The workflow summarizes the structured methodology used in this narrative review. Literature was identified through database searching and supplementary sources, followed by predefined study selection, qualitative data extraction, methodological appraisal, evidence synthesis with confidence grading, and narrative integration within the pharmacological switching framework. Because this review was not conducted as a PRISMA-compliant systematic review or meta-analysis, the figure illustrates the methodological workflow rather than stage-wise counts of studies. Arrows indicate progression through the workflow, whereas colors and icons distinguish the six methodological stages.

This review used a narrative synthesis approach to integrate receptor pharmacology, preclinical disease-model evidence, clinical findings, pharmacokinetic data, and translational barriers for eight minor phytocannabinoids across CNS indications. It was not designed as a Preferred Reporting Items for Systematic Reviews and Meta-Analyses (PRISMA)-compliant systematic review or meta-analysis; rather, its purpose was to develop a mechanistically organized framework for compound-level evaluation, compound–indication matching, and translational prioritization. The overall literature search, study selection, appraisal, and evidence synthesis process is summarized in Figure 1.

Searches were performed in PubMed, Scopus, and Google Scholar for studies published from 1 January 1990 to 10 April 2026. Targeted citation tracking, ClinicalTrials.gov screening for registered trials of minor cannabinoids in neurological or neuropsychiatric indications, and regulatory-source checking were updated through 25 May 2026, the final literature cutoff. Compound-specific search terms included cannabigerol, cannabichromene, cannabinol, cannabidiolic acid, cannabigerolic acid, tetrahydrocannabinolic acid, cannabidivarin, and tetrahydrocannabivarin, together with their corresponding abbreviations. These were combined with indication-specific terms, including epilepsy, neurodegeneration, Huntington’s disease, Parkinson’s disease, Alzheimer’s disease, multiple sclerosis, anxiety, depression, psychosis, autism, pain, substance use, and nausea. Mechanism-specific terms included CB_1_, CB_2_, the vanilloid receptor transient receptor potential vanilloid 1 (TRPV1), the ankyrin receptor transient receptor potential ankyrin 1 (TRPA1), 5-HT_1_A, peroxisome proliferator-activated receptor gamma (PPARγ), G-protein-coupled receptor 55 (GPR55), neuroprotection, and neuroinflammation. Additional studies were identified through forward- and backward-citation tracking of key reviews (Caprioglio et al. [2], Cammà et al. [3], Stone et al. [4], Walsh et al. [5]).

Studies were eligible if they reported pharmacological, preclinical, pharmacokinetic, or clinical data for any of the eight focal compounds at CNS-relevant molecular targets or in neurological, neuropsychiatric, pain, or substance-use models. Eligible study types included in vitro receptor characterization, cellular neuroprotection assays, in vivo disease-model studies, pharmacokinetic investigations, and human clinical or neuroimaging studies. Although the primary search window began in 1990, earlier landmark studies identified through citation tracking were retained when considered essential for historical or mechanistic interpretation.

Studies that focused exclusively on Δ^9^-THC, CBD, or unfractionated cannabis preparations were excluded unless they provided a mechanistic or translational context necessary for interpreting the minor cannabinoid evidence. Semi-synthetic derivatives, including VCE-003, VCE-003.2, and CBDA methyl ester (CBDA-ME), were included when they clarified the pharmacological behavior, target engagement, or translational potential of the parent phytocannabinoid. Peer-reviewed, English-language publications were prioritized; non-peer-reviewed preprints, conference abstracts, and the gray literature were excluded, except for authoritative regulatory documents and clinical-trial registry records used to contextualize regulatory or translational status.

Data were extracted qualitatively for compound identity, chemical class, target engagement, experimental model, dose, route of administration, pharmacokinetic evidence, antagonist validation, sex inclusion, replication status, clinical-development stage, and translational relevance. A single quantitative risk-of-bias instrument was not applied because such tools are designed for systematic reviews of homogeneous outcomes and are poorly suited to a mechanistic synthesis spanning heterogeneous in vitro, preclinical, and early-phase clinical evidence. Each study was instead appraised qualitatively against defined quality dimensions: antagonist or genetic validation of the proposed target, sample size and statistical power, blinding and randomization, sex inclusion, independent replication, and pharmacokinetic confirmation of exposure. Where published formal risk-of-bias assessments were available for a subfield, they were incorporated directly; the neuroprotection literature [4] and the psychiatric [3] literature were each rated at moderate-to-high risk on these criteria, reflecting near-exclusive use of male animals, sparse multi-receptor antagonist controls, and limited blinding and randomization. Conflicting findings were not reconciled by vote-counting or pooling; discrepancies were attributed, where possible, to identifiable methodological or biological sources (differences in dose or receptor occupancy, disease model, assay format, species, or sex), and this attribution is carried into the confidence grade assigned to each mechanistic claim (established, provisional, or proposed; Section 4.4). Publication bias toward positive results, expected in a field dominated by single-laboratory reports and compounds of commercial development interest, was mitigated by grading single-laboratory findings as provisional and flagging the need for independent replication throughout. These limitations are examined in detail in Section 5, Section 6 and Section 8.

## 3. Chemical Diversity and Structure–Activity Relationships

Phytocannabinoid structural diversity originates from a modular biosynthetic logic operating within the glandular trichomes of Cannabis sativa. Two precursor pathways converge: the polyketide route generates olivetolic acid, while the plastidial methylerythritol phosphate pathway supplies geranyl diphosphate [5,23]. An aromatic prenyltransferase, olivetolate geranyltransferase, couples olivetolic acid with geranyl diphosphate to produce CBGA, the universal pentyl (C^5^) precursor. Three dedicated oxidocyclases then channel CBGA toward Δ^9^-THCA, CBDA, and CBCA [1,5]. When divarinolic acid replaces olivetolic acid as the polyketide starter unit, the cascade instead yields the propyl (C^3^) precursor CBGVA and its varinoid derivatives [1]. Non-enzymatic decarboxylation then converts each acidic intermediate to its neutral cannabinoid, and oxidative aromatization of Δ^9^-THC generates CBN [2].

**Figure 2:**
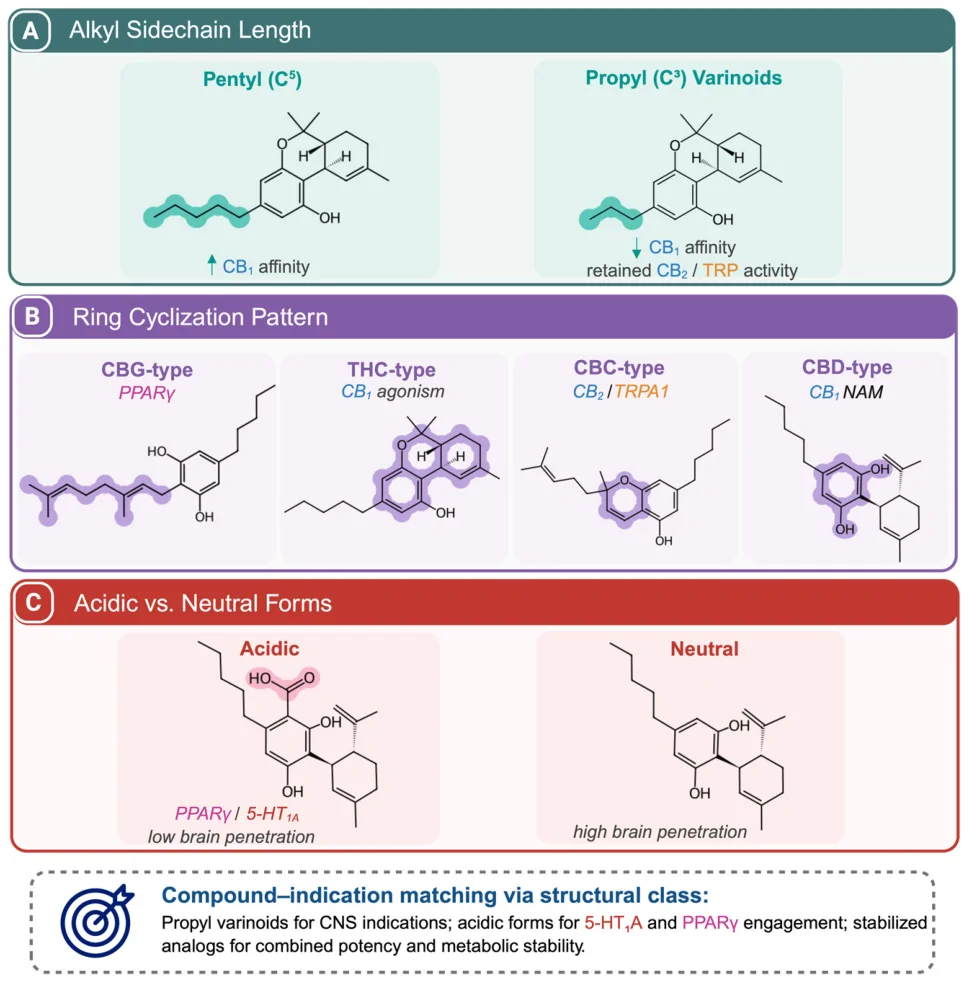
Structural determinants of pharmacological identity in minor phytocannabinoids. Three structural features govern receptor selectivity, signaling efficacy, and CNS access; the distinguishing feature is highlighted in each panel, and target families are distinguished by consistent color coding throughout. (**A**) Alkyl sidechain length: shortening from pentyl (C^5^) to propyl (C^3^; varinoids) lowers CB_1_ affinity while largely retaining CB_2_ and TRP channel activity. (**B**) Ring cyclization sets the principal target: CBG-type favors PPARγ; Δ^9^-THC-type supports orthosteric CB_1_ agonism; CBC-type engages CB_2_ and TRPA1; CBD-type acts as a CB_1_ negative allosteric modulator. (**C**) Oxidation state: acidic forms favor PPARγ and 5-HT_1_A engagement but penetrate the brain poorly (brain-to-plasma ratio < 0.04), whereas decarboxylation to the neutral form increases CNS access. Together, these features support compound–indication matching. Created in BioRender. Bagher, A. (2026) https://BioRender.com/ilv5gw2.

Three structural features collectively govern receptor selectivity, signaling efficacy, and CNS access: alkyl sidechain length, terpenoid ring-cyclization pattern, and oxidation state (acidic versus neutral forms). Their interplay defines the pharmacological identity of each compound and, consequently, its neurotherapeutic potential. These three determinants, and the pharmacological consequences each confers, are summarized in Figure 2. Table 1 catalogs the structural classification and dominant molecular targets of the compounds evaluated in this review. Of these variables, sidechain length exerts the strongest influence on CB_1_ affinity (Figure 2A). A pentyl (C^5^) chain is required to occupy a deep hydrophobic sub-pocket within the CB_1_ orthosteric site [24]. The propyl (C^3^) chain of Δ^9^-THCV is too short to reach this region, accounting for both its lower binding affinity and its predominantly antagonist behavior at CB_1_ [25,26]. The translational significance is considerable: truncation reduces CB_1_-driven psychotropic liability while preserving engagement at CB_2_ and TRP channels. CBDV, for instance, shows greater functional efficacy at CB_2_ than at CB_1_ [27], and TRPV1 and TRPA1 [28], though the extent of this dissociation is compound- and assay-dependent. This window allows anti-neuroinflammatory and anti-excitotoxic pathways to be preferentially engaged while minimizing psychotropic effects.

Ring cyclization represents a second, structurally orthogonal determinant of target selectivity (Figure 2B). CB_1_ activation requires the F200^3.36^/W356^6.48^ toggle-switch motif, a paired rotameric rearrangement first characterized by mutagenesis and homology modeling [29] and later confirmed by crystallographic and cryo-EM analyses of agonist-bound CB_1_ [30,31]. The open-chain geranyl moiety of CBG-type cannabinoids is conformationally unconstrained and unlikely to achieve the pose required for toggle-switch activation, although direct docking of CBG into the CB_1_ orthosteric site has not been reported. Consistent with this, CBG binds PPARγ but does not activate Gi/G-protein-gated inwardly rectifying potassium channel (GIRK) signaling at concentrations up to 20 μM [26,32]. By contrast, the constrained menthanyl ring of Δ^9^-THC-type compounds permits deep orthosteric insertion and engagement of both toggle-switch residues [33]. Chromene ring closure in CBC-type compounds generates a scaffold that activates CB_2_ receptors and gates TRPA1 channels [28,34,35]. In contrast, the resorcinol core of CBD-type compounds favors negative allosteric modulation of CB_1_ [36,37]. Double-bond regiochemistry adds a further layer: shifting the olefin from the Δ^9^ to the Δ^8^ position in THCV approximately doubles the CB_1_-antagonism IC_50_ (Δ^8^-THCV, 757 nM; Δ^9^-THCV, 434 nM), thereby reducing antagonist potency [26].

Acidic cannabinoids introduce a distinct pharmacological dimension (Figure 2C). Retention of the carboxylic acid moiety suppresses CB_1_ agonism and shifts target engagement toward PPARγ (most consistently for CBGA and Δ^9^-THCA) [38] and 5-HT_1_A (most consistently for CBDA) [39], with the relative balance varying by compound. The trade-off is increased molecular polarity, which restricts passive brain penetration, although this limitation is at least partially surmountable through formulation [40]. The broader implication is that receptor selectivity alone is insufficient to predict in vivo neurotherapeutic utility; the interaction among molecular scaffold, target bias, and formulation-dependent CNS access determines which pathways a compound ultimately engages in the intact brain.

**Table 1:** Minor phytocannabinoids evaluated in this review: structural classification, cannabinoid-receptor engagement, dominant CNS-relevant targets with representative affinities, and primary CNS-relevant actions.

Compound	Chemotype/ Sidechain	CB_1_ Engagement	Dominant CNS-Relevant Targets	Representative Affinity (Kᵢ/EC_50_/IC_50_) †	Primary CNS-Relevant Actions
CBG	Neutral (open-chain); C^5^ (pentyl)	None (no GIRK activation at 20 µM)	CB_2_ (weak partial agonist), PPARγ, α_2_-AR (EC_50_ 0.2 nM), 5-HT_1_A (antagonist), TRPA1, Na_V_	CB_1_ Kᵢ ≈ 380 nM; CB_2_ Kᵢ ≈ 150 nM–2.6 µM; α_2_-AR EC_50_ ≈ 0.2 nM; TRPM8 IC_50_ ≈ 160 nM; TRPV1 EC_50_ ≈ 1.3 µM	Anti-neuroinflammatory signaling primarily via PPARγ; arousal and stress-circuit modulation via α_2_-AR agonism; 5-HT_1_A antagonism opposes serotonergic anxiolysis
CBC	Neutral (chromene); C^5^ (pentyl)	None	CB_2_ (agonist), TRPA1 (potent agonist), TRPV1, TRPM8 (functional inactivation)	TRPA1 EC_50_ ≈ 60–90 nM; CB_1_/CB_2_ 10- to 100-fold weaker than Δ^9^-THC	TRP-channel-mediated excitability control; CB_2_-dependent microglial modulation; ERK-dependent neural progenitor support (unconfirmed by antagonist studies)
CBN	Neutral (aromatized THC metabolite); C^5^ (pentyl)	Weak (Kᵢ ≈ 211 nM; potential mild psychoactivity at supratherapeutic doses)	CB_1_ (weak), CB_2_, TRPA1	CB_1_ Kᵢ ≈ 211 nM; TRPV2 EC_50_ ≈ 78 µM	Weak CB_1_/CB_2_ engagement; marketed sedative claims unsupported by controlled evidence; limited neuroprotective signals at supraphysiological concentrations
CBDA	Acidic; C^5^ (pentyl)	None	5-HT_1_A (PAM; ~1000-fold more potent than CBD in 5-HT_1_A [^35^S]GTPγS coupling), TRPV1, TRPA1, PPARγ	5-HT_1_A PAM (no simple Kᵢ); selective COX-2 inhibitor; negligible CB_1_/CB_2_ affinity	Stress-conditional anxiolytic and antiemetic efficacy via 5-HT_1_A allosteric potentiation; anticonvulsant activity in Dravet models; limited by low brain penetration (brain-to-plasma ratio < 0.04)
CBGA	Acidic; C^5^ (pentyl)	None	TRP channels, PPARγ	PPARγ agonist; TRPM8 antagonist; negligible CB_1_/CB_2_ affinity	Bidirectional seizure modulation (anticonvulsant in Dravet, proconvulsant at 6 Hz); exemplifies model-dependent pharmacological switching
Δ^9^-THCA	Acidic; C^5^ (pentyl)	None (~62-fold lower CB_1_ and ~125-fold lower CB_2_ affinity than Δ^9^-THC)	PPARγ (highest transcriptional potency among phytocannabinoids tested), 5-HT_1_A (putative positive modulation), GPR55	CB_1_ Kᵢ ≈ 23.5 nM ‡; PPARγ agonist	PPARγ-dependent neuroprotection with PPARγ-independent IL-6 attenuation (plausibly GPR55-mediated); limited by poor brain penetration and decarboxylation instability
CBDV	Neutral; C^3^ (propyl; varinoid)	None	TRPV1, TRPA1, TRPV4, CB_2_, GPR55, GABA_A_ (putative allosteric modulator)	CB_1_ Kᵢ ≈ 14,700 nM; CB_2_ Kᵢ ≈ 574 nM; TRPV1/TRPA1 agonist (EC_50_ < 10 µM); TRPM8 antagonist	Suppression of neuronal hyperexcitability via convergent TRP desensitization and GABA_A_ potentiation; CB_2_-mediated anti-neuroinflammatory effects
Δ^9^-THCV	Neutral; C^3^ (propyl; varinoid)	Dose-dependent: low-efficacy antagonist (IC_50_ 434 nM); partial agonism at higher exposure (occupancy boundary not defined)	CB_1_ (antagonist → partial agonist), CB_2_, 5-HT_1_A (enhancement, partly orthosteric; active at 100 nM), TRP channels	CB_1_ Kᵢ ≈ 75 nM; CB_2_ Kᵢ ≈ 63 nM	Non-dopaminergic antipsychotic-like profile via CB_1_ antagonism + 5-HT_1_A potentiation; proposed dose-dependent switching from antagonism to partial agonism at CB_1_

† Affinities are binding constants (Kᵢ) or functional potencies (EC_50_/IC_50_) from recombinant or heterologous expression systems and are assay-, species-, and expression-system-dependent, varying substantially across studies; reported affinity does not reliably predict functional potency or the net direction of effect for these compounds. Sources: Refs. [28,39,41,42,43,44] and the systematic human CB_1_/CB_2_ binding study [45]. ‡ The apparent CB_1_ affinity of Δ^9^-THCA is confounded by potential decarboxylation to Δ^9^-THC during handling. AR, adrenoceptor; PAM, positive allosteric modulator.

Eight compounds were selected for detailed evaluation based on five criteria: (i) a non-intoxicating or minimally intoxicating profile, defined by the absence of full CB_1_ agonist efficacy; (ii) documented pharmacological activity at CNS-relevant targets, including CB_2_, TRP channels, 5-HT_1_A, PPARs, or GPR55; (iii) at least one published preclinical neuroprotection study; (iv) natural occurrence in Cannabis sativa; and (v) availability of clinical data, preferred but not mandatory. CBN represents a borderline case under the first criterion: its weak CB_1_ affinity (Kᵢ ≈ 211 nM in rat brain membranes) [43] may produce mild psychoactive effects at supratherapeutic doses. These criteria yielded CBG, CBC, CBN, CBDA, CBGA, Δ^9^-THCA, CBDV, and Δ^9^-THCV, a set that spans C^5^ and C^3^ sidechains, neutral and acidic oxidation states, and four ring-cyclization architectures (Table 1).

Taken together, these structure–activity relationship principles indicate compound–indication matching as a rational design strategy: distinct structural features map onto distinct therapeutic windows. Propyl varinoids (CBDV, Δ^9^-THCV) reduce CB_1_ psychotropic liability while preserving CB_2_ engagement and, in the case of Δ^9^-THCV, adding 5-HT_1_A signaling enhancement [46]. Acidic cannabinoids (CBDA, CBGA, Δ^9^-THCA) shift the receptor profile toward 5-HT_1_A and PPARγ engagement at the expense of brain penetration, a limitation that is surmountable through stabilized analogs such as CBDA-ME [47] or through formulation strategies [40]. Semi-synthetic derivatives such as VCE-003.2 extend these principles by combining receptor selectivity with improved metabolic stability [48].

## 4. Receptor Pharmacology and Molecular Mechanisms

Minor phytocannabinoids act through a distributed network of targets rather than a single dominant receptor [33,49]. This section organizes their molecular pharmacology around four functional axes: presynaptic control of synaptic excitability, neuroinflammatory and microglial regulation, affective and cognitive modulation, and the integrative multi-target behavior linking them, noting for each the receptors engaged, the direction and confidence of the evidence, and where a compound’s dominant mechanism may shift with dose, state, or model.

### 4.1. Modulation of Synaptic Excitability

CB_1_ receptors are densely concentrated at presynaptic terminals across the hippocampus, amygdala, cortex, and cerebellum, where they couple to Gᵢ/G_0_ proteins to suppress adenylyl cyclase, inhibit N-type and P/Q-type Ca^2+^ channels, and activate G-protein-coupled inwardly rectifying potassium (GIRK) channels. The net functional result is a retrograde suppression of neurotransmitter release [5,50,51]. Among the minor cannabinoids tested to date, direct CB_1_ engagement is negligible. None of the minor cannabinoids screened for agonist activity by Walsh and Holmes [26], including CBN, CBG, CBC, and CBDV, elicited significant GIRK activation at 20 μM, well above likely therapeutic plasma exposures, underscoring the absence of meaningful CB_1_ agonist efficacy across the class.

Δ^9^-THCV is the sole exception. It antagonizes CB_1_ with an IC_50_ of 434 ± 24 nM against WIN 55,212-2, and its antagonism is both isomer-dependent (Δ^8^-THCV IC_50_ = 757 nM) and ligand-selective [26]. The structural basis is straightforward: the truncated propyl chain produces a shallower binding pose that stabilizes an inactive CB_1_ conformation at low occupancy, yielding antagonism. At higher concentrations, as receptor occupancy rises, the compound may transition toward partial agonism [27]. This putative dose-dependent switch, in which dose escalation would risk reversing the intended mechanism, underscores the need for Positron Emission Tomography (PET)-based occupancy studies to define the therapeutic window.

The comparison with rimonabant sharpens the translational relevance of this profile. Rimonabant, a CB_1_ inverse agonist, was withdrawn for psychiatric adverse effects plausibly attributed to suppression of constitutive receptor activity [52]. Δ^9^-THCV differs from rimonabant in three respects that may, but do not yet, establish a more favorable safety margin. First, it produced only an incomplete blockade of agonist-evoked Gᵢ/GIRK signaling (maximal inhibition of 79% vs. near-complete inhibition by rimonabant-class inverse agonists), consistent with antagonism rather than inverse agonism, though neutral antagonism has not been directly demonstrated for Δ^9^-THCV in a constitutive-activity assay. Second, its antagonism is ligand-selective rather than global. Third, it concurrently potentiates 5-HT_1_A signaling (see Section 4.3), which may mitigate the mood-destabilizing effects of CB_1_ blockade. Jadoon et al. [53] reported no neuropsychiatric adverse events over 13 weeks in type 2 diabetes, though confirmation in psychiatric populations with longer follow-up is outstanding. Formal classification of Δ^9^-THCV as a neutral antagonist rather than a low-efficacy inverse agonist will require an assay sensitive to constitutive CB_1_ activity, a defined preclinical prerequisite for the psychosis rationale in Section 6.2.

TRP channels provide a mechanistically independent route for controlling excitability. CBDV, CBC, CBDA, and CBG each gate TRPA1 at potencies exceeding the reference agonist mustard oil, though with partial efficacy [28]. Among these, CBDV is the most thoroughly characterized in seizure modulation. It elicits bidirectional TRPV1 currents in HEK293 cells, an effect blocked by capsazepine, and attenuates both the amplitude and the duration of epileptiform bursts in hippocampal slice recordings. Notably, the TRPV1 antagonist 5′-IRTX abolished only the effect on burst duration, indicating that CBDV recruits at least two parallel anti-epileptiform mechanisms, one TRPV1-dependent and the other not [54,55]. CBC occupies a distinct niche within this channel landscape, pairing potent TRPA1 gating with functional inactivation of transient receptor potential melastatin 8 (TRPM8) [28]. These compound-specific channel fingerprints create opportunities for condition-tailored therapeutic design.

Voltage-gated sodium channels (Na_V_) and orphan G-protein-coupled receptors (GPCRs) contribute additional excitability-modulating targets. Hill et al. [56] demonstrated within a single experimental program that CBG (10 μM) blocks Na_V_ currents in SH-SY5Y neuroblastoma cells and mouse cortical neurons, yet fails to attenuate pentylenetetrazole (PTZ)-induced seizures in vivo. This dissociation indicates that Na_V_ blockade alone is insufficient for anticonvulsant efficacy and that multi-target engagement is required at the whole-organism level. Through a separate mechanism, CBDV has been reported to inhibit GPR55, thereby reducing calcium-dependent glutamate release and CNS excitability ([2]). CBDV has also been proposed to modulate γ-aminobutyric acid type A receptor (GABA_A_) receptor signaling, although this mechanism is described primarily in the secondary literature and requires direct primary validation; whether any such activity is mechanistically linked to its engagement of CB_2_ or operates independently likewise remains unresolved [2].

### 4.2. Neuroinflammatory and Microglial Regulation

CB_2_ receptors, expressed predominantly on microglia and astrocytes within the CNS, suppress nuclear factor-κB (NF-κB)-dependent transcription of pro-inflammatory mediators, including tumor necrosis factor-α (TNF-α), interleukin-1β (IL-1β), interleukin-6 (IL-6), inducible nitric oxide synthase (iNOS), and cyclooxygenase-2 (COX-2) [57,58]. Several minor cannabinoids display a preference for CB_2_ over CB_1_: CBC surpassed Δ^9^-THC potency in an AM630-sensitive membrane hyperpolarization assay [34]; CBDV exhibits greater functional efficacy at CB_2_ than at CB_1_ [27]; and CBG acts as a weak partial CB_2_ agonist [59].

Two methodological concerns temper the interpretation of these data. First, the profiles were derived from heterologous overexpression systems in which high receptor density inflates apparent potency via receptor reserve effects. At the lower CB_2_ densities observed in resting human microglia, CBG could, in principle, act as a net antagonist by competing with the endogenous agonist 2-AG. Second, murine microglia exhibit higher basal CB_2_ levels than their human counterparts, and their distribution pattern differs qualitatively. Núñez et al. [60] detected CB_2_ immunoreactivity on perivascular microglia in human brain but not in parenchymal microglia under non-pathological conditions, a restriction not observed in rodent tissue. Because this species difference rests on immunohistochemical evidence from a single human study, it is indicative rather than definitive. This expression-level divergence is compounded by divergence in the receptor itself. Unlike CB_1_, which is approximately 97% identical across humans and rodents, CB_2_ is the least conserved cannabinoid receptor, sharing only about 81 to 82% amino-acid identity between human and rodent orthologues, with the greatest divergence in the C-terminus that governs β-arrestin recruitment and desensitization [61,62]. This produces species-dependent ligand pharmacology: selective CB_2_ agonists can differ by 50- to 140-fold in affinity between rat and human receptors and, in some paradigms, yield opposite functional outcomes across rodent species [61,63]. For minor cannabinoids whose anti-neuroinflammatory effects are attributed in part to CB_2_, rodent efficacy therefore cannot be assumed to predict the direction or magnitude of the human response; CB_2_ engagement should be confirmed at the human receptor, in recombinant human CB_2_ assays, human microglia or induced pluripotent stem cell-derived models, and humanized-receptor mice, before translational claims are advanced. Confirmation in primary human microglia is therefore essential, ideally using both a CB_2_ antagonist (AM630 or SR144528) and a PPARγ antagonist (GW9662 or T0070907) within a single design to dissect the relative contributions of the two receptors. Carrillo-Salinas et al. [64] remains the only study to date to apply both CB_2_ and PPARγ antagonists within a single experimental design, and it stands as the benchmark for mechanistic rigor in this area.

CBC (0.001–1 μM) attenuated lipopolysaccharide (LPS)-induced nitrite accumulation in macrophage cultures [35] and, at 1 μM, promoted neural stem progenitor cell viability via extracellular signal-regulated kinase (ERK)-dependent signaling [65]. However, neither finding has been confirmed with antagonist controls. PPARγ offers a more mechanistically anchored anti-inflammatory pathway. Activation of this nuclear receptor transcriptionally silences NF-κB in microglia and astrocytes [66]. CBG, Δ^9^-THCA, and CBDA each activate PPARγ, with Δ^9^-THCA exhibiting the highest transcriptional potency among the phytocannabinoids tested [38]. CBG and CBDA also act as dual PPARα/γ agonists, with downstream effects on lipid metabolism [67]. The approximately 62-fold lower CB_1_ and 125-fold lower CB_2_ affinity of Δ^9^-THCA relative to Δ^9^-THC [68] is consistent with predominantly PPARγ-mediated effects. However, the chemical instability of Δ^9^-THCA and its facile decarboxylation to the psychoactive Δ^9^-THC may confound attribution in any study that does not verify analyte integrity [4,40].

The semi-synthetic CBG derivatives VCE-003 and VCE-003.2 illustrate the separability of CB_2_ and PPARγ contributions. VCE-003, a CBG-derived aminoquinone, retains dual CB_2_ and PPARγ agonist activity; accordingly, Carrillo-Salinas et al. [64] showed that its suppression of cytokine secretion in primary microglia was attenuated by both AM630 (CB_2_) and GW9662 (PPARγ). VCE-003.2, a further structural modification engineered to eliminate cannabinoid receptor engagement while preserving PPARγ selectivity, activates PPARγ (IC_50_ 1.2 μM) with negligible CB_1_/CB_2_ binding (Kᵢ > 40 μM; [69,70]. That VCE-003.2 retains neuroprotective efficacy across multiple disease models (Section 5.2) despite lacking CB_2_ activity shows that PPARγ activation alone can drive substantial anti-neuroinflammatory effects, though whether this suffices without CB_2_ co-activation in all contexts remains open. GPR55 constitutes a further immunomodulatory node: Δ^9^-THCA-mediated reduction in interleukin-8 (IL-8) secretion was abolished by the GPR55 antagonist CID16020046 but was unaffected by CB_1_/CB_2_ blockade [71]. In each of these contexts, the therapeutic objective is not wholesale microglial suppression but phenotypic recalibration toward a less neurotoxic activation state [72,73].

### 4.3. Affective and Cognitive Modulation

The 5-HT_1_A receptor serves as a key convergence point for affective modulation by minor cannabinoids. CBDA is the best-supported 5-HT_1_A positive allosteric modulator among the minor cannabinoids discussed here, amplifying endogenous serotonin signaling rather than competing for the orthosteric site [39]. Δ^9^-THCV appears to enhance 5-HT_1_A receptor signaling through a more complex profile that may combine positive modulation with partial orthosteric-displacement behavior [46], and the evidence for Δ^9^-THCA as a classical 5-HT_1_A PAM remains less definitive [2]. CBDA stands out for its potency: in a [^35^S]GTPγS binding assay using rat brainstem membranes, it enhanced 8-hydroxy-2-(di-n-propylamino)tetralin (8-OH-DPAT)-stimulated G-protein activation at concentrations approximately 1000-fold lower than those of CBD [39]. This reflects allosteric amplification of agonist-driven coupling, not intrinsically 1000-fold greater anxiolytic efficacy. The magnitude of separation varies across assay systems: approximately 1000-fold in [^35^S]GTPγS coupling at 5-HT_1_A and in conditioned gaping models of nausea [39,74], but only 10- to 100-fold in anticonvulsant assays [40], where additional non-serotonergic mechanisms contribute to the efficacy of both compounds. Moreover, the in vitro advantage is counterbalanced by limited brain penetration and a short plasma half-life. Δ^9^-THCV, by contrast, potentiates 5-HT_1_A at concentrations as low as 100 nM [46] with substantially better CNS penetration. CBG diverges sharply: it competitively antagonizes 5-HT_1_A [42], a divergence that likely underpins the distinct anxiety-paradigm profiles across these compounds.

CBG’s receptor profile is further complicated by its potent α_2_-adrenoceptor agonism (EC_50_ 0.2 nM in heterologous expression systems), a value that likely overestimates functional potency at native receptor densities on locus coeruleus projection neurons [42]. Its 5-HT_1_A antagonism directly opposes the serotonergic anxiolysis of CBDA and Δ^9^-THCV, while its α_2_-adrenergic activity may independently modulate arousal and stress reactivity via presynaptic inhibition of norepinephrine release in locus coeruleus circuits. At low doses, α_2_-mediated sedation could produce apparent anxiolytic-like effects in locomotor-dependent assays such as the open field, whereas concurrent 5-HT_1_A blockade would be expected to oppose anxiolysis in serotonin-dependent paradigms such as the elevated plus maze under stress-primed conditions. The net behavioral output, therefore, depends on the relative engagement of each target, a balance that likely shifts with dose, stress state, and brain region, and that cannot be resolved without dual-antagonist studies combining yohimbine (α_2_) and WAY-100635 (5-HT_1_A). This competing pharmacological account provides a parsimonious explanation for CBG’s inconsistent outcomes in anxiety-related paradigms [3] and argues against positioning CBG as either an anxiolytic or anxiogenic compound without specifying the paradigm, dose, and stress context.

### 4.4. Integration: Multi-Target Pharmacology as Opportunity and Interpretive Challenge

The neurotherapeutic promise of minor cannabinoids lies not in high-affinity action at any single receptor but in the concurrent engagement of multiple signaling systems. This polypharmacological logic is well established for CNS disorders, whose polygenic origins and circuit-level complexity have long favored non-selective agents over single-target ligands [75,76], and it has recently been articulated for CBD across neuropsychiatric indications [77]. Δ^9^-THCV exemplifies this: it may simultaneously antagonize CB_1_, modulate CB_2_, and potentiate 5-HT_1_A, each within distinct circuits yet converging on shared pathological endpoints [6,42,46]. Such breadth is well matched to the multifactorial nature of neurological disease, but the net consequence of multi-target engagement varies not only between compounds but within a single compound across dose, physiological state, and disease model. The pharmacological switching framework developed here offers a falsifiable means of classifying that variability (Box 1).

The same breadth that creates this interpretive problem also carries therapeutic and developmental liabilities. Broad target engagement reduces selectivity, so intended actions are accompanied by potentially unwanted ones, and the effect profile is harder to predict than for a selective ligand. It also complicates dose optimization: because the engaged targets differ in affinity by orders of magnitude, no single dose optimally engages all of them, and a dose tuned to one target may sub- or supra-optimally engage another. The switching phenomenon sharpens this: a dose that crosses an occupancy or state boundary can reverse rather than merely weaken the intended effect, making the therapeutic window not simply narrow but context-dependent. Together with the drug–drug interaction risk of promiscuous CYP inhibition (Section 7.3) and the difficulty of attributing a clinical effect to a single mechanism (Section 7.4), these liabilities are addressed here through biomarker-guided, occupancy-informed dosing.

Box 1 **Operational definition and classification criteria for pharmacological switching.**

Pharmacological switching denotes a dose-, state-, or model-dependent shift in the dominant determinant of a compound’s net effect that reverses the direction, rather than merely the magnitude, of that effect on a defined functional endpoint.

An effect is classified as switching only when all of the following are met:

1. Directional reversal: The sign of the net outcome changes (e.g., anticonvulsant to proconvulsant; or, hypothetically, CB_1_ antagonism with antipsychotic-like valence to partial agonism with psychotomimetic-like valence), not merely the potency or magnitude of a unidirectional effect.
2. Mechanistic attribution: The reversal is traceable to a shift in which receptor system predominates or in the efficacy mode expressed at a single receptor, rather than to a non-specific or off-target action. Predominance is defined operationally as the target whose selective blockade or genetic deletion abolishes the larger fraction of the effect under the condition tested, and that fraction should be reported; where no such manipulation has been performed, the attribution is inferred, and the instance cannot exceed the provisional tier.
3. Boundary specification: The dose, state, or model boundary at which the reversal occurs is defined a priori, so the claim is confirmed by a demonstrated reversal at that boundary and refuted by its absence.
4. Validation where available: The reversal is corroborated by receptor antagonism, genetic deletion or knockdown, or selective tool compounds, rather than inferred from the net phenotype alone.
5. Exclusion of alternative explanations: The reversal is not adequately attributable to experimental variability (noise, underpowering, or non-reproducibility) or to unmatched model biology (baseline differences between paradigms that would yield divergent outcomes for any intervention). A reversal that co-varies with a study-level confounder, is not reproducible under the same boundary condition, or cannot be recreated within a single model by titrating the controlling variable, is classified as variability or model difference rather than switching.

Explicitly excluded: monotonic potency or efficacy changes that preserve the direction of effect; graded antagonist–agonist transitions that do not alter behavioral valence; pathway-selective (biased) signaling that redistributes downstream coupling without reversing net direction; and hormetic (biphasic) responses, whose reversal reflects a single mechanism’s concentration–response shape rather than a shift in the dominant mechanism (criterion 2).

An instance meeting criteria 1–4 but not fully resolvable under criterion 5 is graded proposed rather than established or provisional. An effect that emerges only under a permissive state, being absent rather than reversed under basal conditions, is recorded as a partial reversal and graded on the same scale.

Switching is not a new phenomenon but a formalization of familiar ones: biphasic or hormetic dose–response [78,79], state-dependent drug action [80], and model-dependent efficacy [81,82], under a single, endpoint-anchored criterion: reversal of the net direction of effect. This criterion is what separates switching from the concepts most readily conflated with it: functional selectivity (biased agonism) [83,84], hormesis, and context-dependent pharmacology, each of which alters the magnitude or downstream distribution of a response without requiring a change in its direction; hormesis can reverse direction, but only as the biphasic dose–response of a single mechanism rather than through a change in which mechanism dominates (Table 2). Context-dependent pharmacology is therefore the superordinate category rather than a parallel one: switching is the subset of context-dependent effects in which a change in context reverses the direction of the net effect, and that reversal is traceable to a change in which mechanism dominates. Biased agonism is complementary rather than competitive: Δ^9^-THCV’s occupancy-dependent transition would be an intra-target efficacy switch that may coexist with G protein- versus β-arrestin- biased recruitment at the same receptor.

Two constructs from classical receptor theory, low-efficacy partial agonism and protean agonism, would account for Δ^9^-THCV’s antagonism-to-agonism transition without invoking a new framework, and are compared in Table 2 [85,86]. Switching differs from both in being anchored to the net effect on a defined functional endpoint, in admitting reversals in which dominance moves between distinct receptor systems, and in requiring the controlling variable and its boundary to be specified in advance; the dose-dependent mode is accordingly the limiting case in which it converges on established single-receptor behavior.

**Figure 3:**
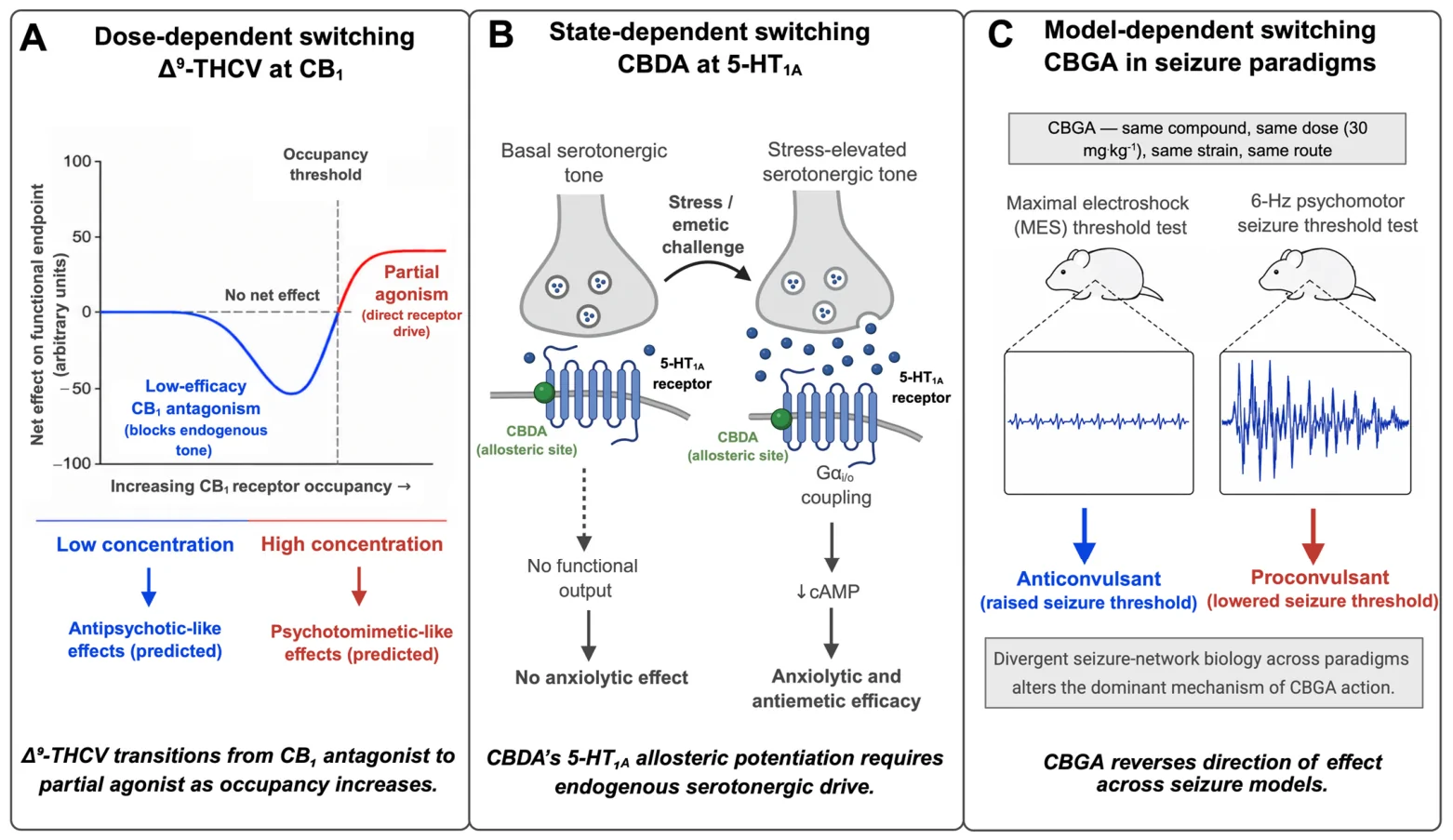
Three modes of pharmacological switching in minor phytocannabinoids. Switching is defined as a dose-, state-, or model-dependent shift in the dominant determinant of a compound’s net effect that reverses the direction, rather than merely the magnitude, of that effect on a defined functional endpoint. Panels (**A**–**C**) show one candidate instance per mode; none currently satisfies all five criteria in Box 1. (**A**) Dose-dependent. Δ^9^-THCV acts as a low-efficacy CB_1_ antagonist at low receptor occupancy and transitions to partial agonism above a threshold, so the net effect reverses sign rather than merely diminishing; the predicted shift from antipsychotic-like to psychotomimetic-like outcomes has not been demonstrated behaviorally. The biphasic profile assumes that the antagonist and agonist modes differ in concentration dependence, which has not been experimentally established. (**B**) State-dependent. CBDA potentiates 5-HT_1_A allosterically, producing no measurable effect under basal conditions but anxiolytic and antiemetic efficacy when serotonergic drive is mobilized by stress or emetic challenge. (**C**) Model-dependent. CBGA is anticonvulsant in the maximal electroshock threshold test, yet proconvulsant in the 6-Hz psychomotor threshold test in Swiss male mice at the same dose (30 mg·kg^−1^), route, and pretreatment interval, excluding species, strain, and exposure as explanations. Switching is distinct from biased agonism, which redistributes downstream coupling without reversing net direction. Arrows indicate the direction of the predicted or observed functional outcome, whereas blue denotes anticonvulsant/antagonist-like or lower-concentration effects and red denotes proconvulsant/partial-agonist-like or higher-concentration effects. Created in BioRender. Bagher, A. (2026) https://BioRender.com/iui5dg3.

Three operationally distinct modes are discernible in the current evidence (Figure 3), each illustrated by a single worked case resting on one or two, frequently single-laboratory, studies; the generality of switching across the class should be regarded as a hypothesis for systematic testing. The dose-dependent mode is motivated by Δ^9^-THCV, whose CB_1_ antagonism is reported to give way to partial agonism as receptor occupancy rises [26,27]; because the antagonism and the agonist-like effects were established in separate systems and no common functional endpoint has been titrated across the transition, this instance does not yet satisfy criterion 1 and is retained as the case that motivates the framework rather than one that validates it. State-dependent switching is illustrated by CBDA, whose anxiolytic effect emerges only under stress-primed conditions that mobilize the serotonergic tone required for allosteric potentiation [87]. The model-dependent mode, exemplified by CBGA, is anticonvulsant in the *Scn1a^+/−^* paradigm yet proconvulsant in the 6-Hz assay at equivalent doses [88,89], the case most susceptible to the objection that a cross-paradigm reversal reflects unmatched model biology. It meets the Box 1 criteria only conditionally and is classified as proposed, contingent on demonstrating that the reversal tracks a measurable difference in endocannabinoid tone rather than incidental model divergence or GABAA modulation with receptor desensitization [88,89]. When these patterns are further compounded by sex-dependent dose requirements [90,91] and antagonistic cannabinoid–cannabinoid interactions [53], monotonic dose–response assumptions break down.

The framework is deliberately disconfirmable. It predicts that a positron emission tomography–defined CB_1_ occupancy ceiling, rather than administered dose, should demarcate the antipsychotic-like from the psychotomimetic-like direction of Δ^9^-THCV (Section 6.2); that CBDA anxiolysis requires demonstrable serotonergic mobilization; and that CBGA’s directional reversal tracks a measurable difference in endocannabinoid tone between paradigms. Failure to observe any of these boundaries would refute the corresponding switching claim, the property that separates the framework from a post hoc redescription of context-dependent effects.

A cross-cutting feature reinforces this framework: several of the most therapeutically consequential interactions in the class are allosteric. CBDA potentiates 5-HT_1_A activation through an allosteric mechanism [39]; Δ^9^-THCV likewise potentiates 8-OH-DPAT-stimulated 5-HT_1_A signaling but also partially displaces the orthosteric radioligand, indicating a partly orthosteric action [42]; CBDV is proposed, largely on the basis of the secondary literature, to modulate GABA_A_ allosterically [2]; and CBD operates as a negative allosteric modulator at CB_1_ (Section 3). Allosteric engagement confers properties that orthosteric agonism lacks and that are well established in the GPCR literature and specifically advanced for CNS targets [92,93]: an intrinsic ceiling on maximal effect that limits overdose risk, signal-dependent amplification that accounts for the stress-conditionality of CBDA’s action, resistance to receptor desensitization, and a response that tracks endogenous transmitter tone. This property is at once a precision-medicine asset amenable to biomarker stratification and a source of unpredictable heterogeneity in unstratified populations. Exploited deliberately, it would reposition several minor cannabinoids as precision amplifiers of endogenous neurotransmission rather than pharmacological overrides.

Table 3 summarizes the documented instances of pharmacological switching and their translational implications. With the receptor pharmacology and this integrative framework now established, the review turns to how these signaling signatures translate, or fail to translate, into preclinical efficacy across CNS disease domains.

## 5. Preclinical Evidence Across CNS Indications

This section evaluates how the receptor signatures cataloged above translate into preclinical efficacy, organized by disease domain. Several signals are derived from single-laboratory studies with limited mechanistic interrogation. Table 4 presents a compound-by-indication signal matrix annotating effect direction, replication status, and mechanistic confidence for all eight compounds across thirteen CNS indications. Table 5 supplements this overview with model context, mechanistic attribution, and key references for each non-null finding. One cross-cutting concern applies throughout: formal risk-of-bias assessments identified moderate-to-high risk across both the psychiatric literature [3] and the neuroprotection literature [4], with near-exclusive use of male subjects, sparse multi-receptor antagonist controls, and limited blinding and randomization, all of which constrain projection of preclinical efficacy onto clinical populations.

### 5.1. Epilepsy and Neuronal Hyperexcitability

Roughly one-third of patients with epilepsy remain refractory to available antiseizure medications [2]. The most comprehensive recent synthesis of the cannabinoid–epilepsy literature has been provided by Devinsky et al. [124] in Physiological Reviews. It covers the dominant evidence on Δ^9^-THC and CBD across acute seizure models, chronic epileptogenesis, and randomized controlled trials, and concludes that the anticonvulsant mechanism of CBD remains incompletely resolved despite regulatory approval for Dravet syndrome, Lennox–Gastaut syndrome, and tuberous sclerosis complex [124]. The central question here is therefore not whether minor cannabinoids engage seizure-relevant targets (as the preceding section established), but whether their profiles translate into reproducible anticonvulsant efficacy at exposures distinct from those in the broader cannabinoid–epilepsy evidence base reviewed by Devinsky et al. [124]. This section focuses on the adjacent minor-cannabinoid evidence, which has not yet been systematically integrated.

CBDV remains the best-characterized compound. CBDV (200 mg·kg^−1^ i.p.) rendered 90% of DBA/2 mice seizure-free in the audiogenic model and reduced PTZ-induced seizure severity in rats [54]. A CBDV-rich botanical extract reproduced these effects via a CB_1_-independent mechanism [95], and oral CBDV (400 mg·kg^−1^) similarly suppressed PTZ seizures [125]. However, CBDV was ineffective against pilocarpine-induced seizures unless co-administered with valproate or phenobarbital [54,95]. This model-dependent profile is mechanistically instructive: CBDV’s TRPV1 modulation and putative GABA_A_-receptor potentiation appear sufficient to dampen the neuronal hyperexcitability underlying the audiogenic and pentylenetetrazole models, the latter arising from blockade of GABA_A_-mediated inhibition, but insufficient against the broader, self-sustaining excitotoxic cascade of pilocarpine-induced status epilepticus.

CBDA increased the seizure temperature threshold in the Scn1a^+/−^ Dravet model at 10–30 mg·kg^−1^, doses substantially lower than those reported for CBD [40]. Clobazam potentiation in this model has been demonstrated for CBGA, but not for CBDA [88]. This signal remains single-laboratory, with antagonist-controlled mechanistic attribution at TRPV1 and 5-HT_1_A but without inter-laboratory replication. Stabilized CBDA-enriched hemp extracts also produced dose-dependent anticonvulsant protection in the maximal electroshock (MES) test, with an entourage-like effect (multi-constituent enhancement) when additional minor cannabinoid constituents were present [109]. Potency comparisons with CBD should remain paradigm-restricted, given the confounding influence of formulation, route, and model on apparent rank order.

Δ^9^-THCV produced anticonvulsant activity in PTZ models at 0.25 mg·kg^−1^ i.p., yielding complete seizure absence in 33% of animals [96]. In the Dravet thermal-threshold assay, however, Δ^9^-THCV (3 mg·kg^−1^) was proconvulsant and lowered the seizure threshold [88]. The compound therefore displays model-dependent bidirectionality in seizure paradigms, resembling CBGA and supporting its classification as a mixed rather than uniformly anticonvulsant signal, although the 12-fold dose difference between paradigms precludes attributing the reversal to model identity (Table 3). The absence of antagonist studies precludes target attribution, particularly given THCV’s context-dependent CB_1_.

CBGA exemplifies the bidirectional profile that defines model-dependent pharmacological switching (Section 4.4): anticonvulsant in the Dravet hyperthermia paradigm and potentiates clobazam, and anticonvulsant in the maximal electroshock threshold test, yet proconvulsant in the 6-Hz threshold test at the same dose (30 mg·kg^−1^), in the same mouse strain, by the same route and pretreatment interval, and proconvulsant against spontaneous seizures at the higher of two doses [88]; this directional reversal may reflect model-specific endocannabinoid tone or, alternatively, context-dependent GABAergic modulation with receptor desensitization [89]. CBG failed to reduce PTZ-induced seizure severity despite confirmed Na_V_ blockade in vitro [56], and CBC was inactive in the MES test [103].

Despite this preclinical promise, CBDV did not separate from placebo in a Phase II epilepsy trial (discussed in Section 6) and also failed to separate from placebo in human immunodeficiency virus (HIV)-associated neuropathic pain [126]. CBDV and CBDA remain the most credible anticonvulsant candidates in this class, although CBDA’s poor brain penetration (brain-to-plasma ratio < 0.04) and its liability to decarboxylate to CBD constrain the parent acid and argue for stabilized analogs such as CBDA-ME or penetration-enhancing formulations; both compounds also require cross-model replication and better alignment of dose and target population.

Summary: The best evidence is for CBDV (CB_1_-independent efficacy across audiogenic and PTZ models, independently reproduced) and CBDA (Dravet threshold elevation with antagonist-controlled TRPV1/5-HT_1_A attribution), though the CBDA signal is single-laboratory, and Δ^9^-THCV and CBGA show proconvulsant reversals in some paradigms. CBDV and CBDA are the most credible candidates but require cross-model replication and paradigm-matched development, with seizure-liability monitoring warranted given the documented bidirectionality. Against Box 1, this domain contains the only instance in the review that meets criterion 1: the CBGA anticonvulsant-to-proconvulsant reversal, graded proposed because the mechanistic attribution is inferred and the controlling variable was not specified in advance (Table 3).

### 5.2. Neurodegeneration and Central Neuroinflammation

Minor cannabinoids and their derivatives warrant evaluation as disease-modifying agents in neurodegeneration, but the evidence remains preclinical and is strongest for PPARγ-linked modulation of neuroinflammation rather than confirmed slowing of human disease progression. Three compounds (Δ^9^-THCA, VCE-003, and VCE-003.2) have antagonist-validated PPARγ engagement in HD, MS, and PD, respectively, making this the most internally consistent mechanistic signal in the class; the PPARγ linkage proposed for CBG, and for VCE-003.2 in PD and amyotrophic lateral sclerosis (ALS), remains inferred rather than antagonist-confirmed. This convergence must, however, be read against a methodological asymmetry: Stone et al. [4] noted that most in vivo neuroprotection studies of minor cannabinoids tested only PPARγ. The apparent prominence of PPARγ may therefore partly reflect selective deployment of T0070907 or GW9662 rather than the absence of parallel CB_2_, GPR55, or nuclear factor erythroid 2–related factor 2 (Nrf2)-independent contributions, which the available designs did not rule out. The Δ^9^-THCA HD data (below) illustrate this: PPARγ blockade attenuated most but not all anti-inflammatory effects, and IL-6 reduction persisted, implicating an additional, plausibly GPR55-mediated mechanism. PPARγ is therefore best regarded as the most consistently detected mechanism rather than the exclusive one.

Even where neuroprotection is confirmed by antagonists as PPARγ-dependent, it is not the output of a single linear pathway. As a ligand-activated transcription factor, PPARγ functions as a convergence hub: its activation transrepresses NF-κB to lower pro-inflammatory mediators (TNF-α, IL-1β, iNOS, COX-2), induces Nrf2-coordinated antioxidant defenses, supports mitochondrial bioenergetics, and promotes a shift in microglia toward a reparative phenotype. For the minor cannabinoids, the documented effects (Nrf2 restoration and reductions in COX-2, iNOS, and cytokine levels [99,100] are consistent with this multi-arm output, although not every arm has been dissected for every compound. PPARγ-mediated neuroprotection is thus an integrated downstream network rather than a single effector, operating alongside the parallel contributions of CB_2_ and GPR55 noted above. Disease by disease, the evidence varies markedly, from a single in vitro screening study for AD to multi-compound, multi-model convergence in HD.

Alzheimer’s disease: Support comes primarily from Schubert et al. [97]. CBG (100 nM) prevented amyloid-β accumulation in MC65 neurons, preserved trophic factors in cortical neurons (EC_50_ 1.5 μM), and prevented oxytosis in HT22 cells (EC_50_ 1.9 μM). Schubert et al. [97] reported that neither MC65 nor HT22 cells express CB_1_ or CB_2_ receptors, leading the authors to conclude that these neuroprotective effects are cannabinoid receptor-independent [97]. This conclusion is consistent with, but not definitively attributable to, the PPARγ engagement and Nrf2 restoration demonstrated for CBG in neuroinflammatory contexts [99,100]. Whether the absence of CB_1_/CB_2_ was confirmed by expression profiling or inferred from prior characterization is not specified. Notably, a subsequent study demonstrated functional CB_1_ expression in HT22 cells using pharmacological antagonism (AM251) and CB_1_-siRNA knockdown [98], suggesting cannabinoid receptor-independent neuroprotection may not be the sole mechanism in this line. Independent verification of the receptor status in MC65 cells remains outstanding.

Parkinson’s disease: VCE-003.2 reduced nigrostriatal damage and neuroinflammation in LPS-lesioned mice, with the glial and cytokine responses attenuated by the PPARγ antagonist T0070907, although the corresponding cell-based effects were not antagonist-sensitive [121]. Oral dosing, however, produced only modest functional recovery at the highest dose tested [48]. Δ^9^-THCV preserved dopaminergic neurons in both the 6-hydroxydopamine (6-OHDA) and LPS models [118], but CB_2_ attribution remains inferential without same-design antagonist experiments. The dataset lacks cross-laboratory replication and chronic progressive models.

Huntington’s disease: This indication provides the most coherent case for neuroprotection. CBG improved multiple readouts in the 3-nitropropionate model while activating PPARγ in striatal cells [99]. VCE-003.2 improved neuronal survival and motor performance [69] and promoted neurogenesis in mutant huntingtin models [122]. Δ^9^-THCA improved hindlimb dystonia, locomotor activity, and inflammatory markers in the 3-nitropropionate model; co-administration of T0070907 blocked most of these benefits, including improvements in motor function, astrogliosis, and microgliosis, and attenuation of TNF-α, iNOS, and COX-2, but did not reverse IL-6 reduction [38]. The PPARγ-resistant IL-6 attenuation suggests Δ^9^-THCA engages at least one additional, plausibly GPR55-mediated, anti-inflammatory mechanism [71]. The convergence of PPARγ-dependent neuroprotection across distinct compounds and models supports this axis as a genuine mechanistic theme (though, as noted, its apparent exclusivity is conditioned by the antagonist-selection patterns of the source literature); the field remains at the stage of plausibility rather than translational maturity.

Multiple sclerosis: VCE-003 reduced clinical severity, demyelination, and axonal injury in experimental autoimmune encephalomyelitis (EAE), and these effects were blocked by both CB_2_ and PPARγ antagonists [64]. A similar benefit was observed in Theiler’s murine encephalomyelitis virus (TMEV)-induced demyelination [120]. This narrow compound representation prevents broader class-level generalization.

Amyotrophic lateral sclerosis and neuroregeneration: VCE-003.2 preserved spinal motor neurons and reduced astrogliosis in superoxide dismutase 1 (SOD1)-G93A mice, with motor benefits that were partial and not sustained to the 18-week endpoint [70]. CBN delayed symptom onset without altering survival [106]. Outside specific disease models, CBG (2.5–7.5 μM) uniquely restored Nrf2 levels depleted by inflammatory insult [100], and CBC (1 μM) promoted neural stem progenitor viability via ERK signaling while suppressing astroglial differentiation [65]. Both findings lack confirmation by antagonists and in vivo replication. ALS and neuroregeneration remain exploratory indications for this class.

One observation about the overall evidence base deserves emphasis. VCE-003.2’s candidacy as the strongest semi-synthetic neuroprotective candidate rests on a coherent multi-model dataset spanning PD, HD, and ALS preparations, antagonist-validated in HD and partially so in PD, complemented by evidence from MS for the parent compound, VCE-003. It also represents the clearest case of rational, mechanism-led optimization in this review: targeted modification of a natural scaffold to remove cannabinoid-receptor engagement while concentrating PPARγ activity converts the multi-target ambiguity of the parent phytocannabinoids into a single, pharmacologically defined, antagonist-confirmed mechanism. Coupled with its improved metabolic stability, this makes VCE-003.2 the most mechanistically tractable neuroprotective candidate in the class, notwithstanding its still-preclinical status. The internal consistency of this evidence is notable, but the dataset derives predominantly from a single Spanish research consortium led by Fernández-Ruiz and Muñoz [48,64,70,121,122]; independent inter-laboratory replication remains the principal outstanding requirement for further translational confidence in this candidate.

Summary: The most consistent evidence is the PPARγ anti-neuroinflammatory signal shared by CBG, Δ^9^-THCA, VCE-003, and VCE-003.2 across HD, PD, MS, and ALS models, with antagonism validated in HD and MS and inferred elsewhere, but all data are preclinical. PPARγ prominence is partly attributable to selective antagonist use, and no study has shown slowed human disease progression. VCE-003.2 is the lead candidate, contingent on human-tissue confirmation (given the CB_2_ species divergence in Section 4.2) and biomarker-guided trials in progressive-disease models. Against Box 1, no instance in this domain meets criterion 1. The closest candidate, Δ^9^-THCV in Parkinson’s models, shows a shift in dominant mechanism between 6-OHDA and LPS lesions: antioxidant and CB_2_-independent in the former, and CB_2_-attributed in the latter, without any reversal in the direction of the neuroprotective effect; therefore, it qualifies as context-dependent pharmacology rather than a switch.

### 5.3. Mood, Anxiety, and Cognition

The psychiatric literature is broader in behavioral scope but less mechanistic in depth than the preceding domains. Cammà et al. [3] synthesized 22 preclinical studies and one clinical study, highlighting both promise and substantial heterogeneity. This body of work is signal-generating rather than treatment-defining.

Anxiety: CBDA and its stabilized methyl ester, CBDA-ME, exhibit the most consistent anxiolytic profiles within this class. CBDA (0.1–100 μg·kg^−1^ i.p.) attenuated stress-induced anxiogenic behavior in the light–dark emergence test in rats previously exposed to footshock [87], and replicated this effect at doses of 0.1–30 mg·kg^−1^ [94]. CBDA-ME reproduced the anxiolytic profile at doses as low as 0.01 μg·kg^−1^. Critically, CBDA did not modify anxiety-like behavior in unstressed animals and yielded inconsistent results across the open field, elevated plus maze, and novelty-suppressed feeding tests [3,113,127]. This conditional efficacy is consistent with the requirement for elevated serotonergic tone for 5-HT_1_A allosteric potentiation, but has not been directly demonstrated in vivo. CBG has been consistently negative in anxiolytic assays [101,102], plausibly reflecting the opposing actions of its 5-HT_1_A antagonism and α_2_-adrenergic agonism.

Depression: CBDA-ME showed antidepressant-like effects in the forced swim test with a pronounced sex-dependent dose differential [90,91]. CBC reduced immobility in both the forced swim and tail suspension tests, though only modestly [104]. Both findings warrant conservative interpretation, given the well-recognized limitations of the acute forced swim test as an antidepressant proxy.

Psychosis: Δ^9^-THCV data in phencyclidine models are among the strongest psychiatric findings, spanning positive, negative, and cognition-relevant symptom domains [3,46]. These are preclinical findings, however: no controlled human trial has tested Δ^9^-THCV in psychosis, and the therapeutic rationale therefore rests on receptor pharmacology and animal-model data rather than demonstrated clinical efficacy. The proposed 5-HT_1_A mediation additionally requires validation with antagonists. CBDA failed to attenuate methamphetamine-induced hyperlocomotion [110], defining a mechanistic boundary: serotonergic allosteric modulation appears insufficient to counteract dopaminergic reinforcement in this circuit.

Neurodevelopmental disorders: CBDV improved autism spectrum disorder (ASD)-like behavioral outcomes in valproate-exposed rodents [117] and extended survival in methyl-CpG-binding protein 2 (MeCP2)-mutant Rett syndrome mice [116]. Each of these findings derives from a single laboratory; replication and convergent neuroimaging evidence in humans [128,129], discussed in detail in the clinical evidence section, support the translational hypothesis, but a Phase IIa efficacy trial in pediatric ASD has not yet been reported [130]. The single-dose neuroimaging findings constitute proof of mechanism-based target engagement, not evidence of symptomatic benefit.

Nausea and emesis: CBDA’s antiemetic effects represent the clearest pharmacology-to-phenotype match in this review. In preclinical conditioned gaping models, CBDA is approximately 1000-fold more potent than CBD, effective against both acute and anticipatory nausea [74,131], and synergistic with ondansetron at low doses [111]. Whether this preclinical potency advantage translates proportionally to human antiemetic efficacy remains to be established.

Across this domain, CBDA appears most promising for stress-sensitive anxiety and nausea, whereas CBDV has the better translational bridge to neurodevelopmental disorders, given its existing human neurobiological data.

Summary: The strongest signals are for CBDA/CBDA-ME as stress-conditional anxiolytics and CBDA’s antiemetic activity (the clearest pharmacology-to-phenotype match here), alongside Δ^9^-THCV in psychosis models and CBDV in neurodevelopmental models, though these studies are behaviorally broad but mechanistically shallow, largely single-laboratory, and awaiting antagonist validation. CBDA is most promising for stress-sensitive anxiety and nausea, whereas CBDV offers a better translational bridge into neurodevelopmental disorders, given its human neuroimaging data, pending the unreported Phase IIa readout. Against Box 1, this domain contains two non-model-dependent candidates, but neither fully qualifies: CBDA’s stress-conditional anxiolysis is a state-conditional emergence of effect rather than a change in sign, and is graded provisional, while the predicted transition of Δ^9^-THCV from antipsychotic-like to psychotomimetic-like valence has not been demonstrated on a common functional endpoint, and is graded proposed (Table 3).

### 5.4. Pain and Substance Use Disorders

Pain and substance use share a common evidentiary limitation: the minor cannabinoid literature in both domains is dominated by single-laboratory findings restricted to narrow experimental paradigms. Neither domain yet supports therapeutic claims beyond signal generation.

Pain is the most frequently cited reason for medical cannabinoid use, yet the minor-cannabinoid literature is confined almost entirely to acute inflammatory models. Neuropathic, visceral, and cancer pain remain effectively untested. Δ^9^-THCV reduced carrageenan- and formalin-induced pain behaviors and was sensitive to both CB_1_ and CB_2_ antagonists [119], illustrating the context dependence of THCV’s pharmacology rather than contradicting its in vitro receptor classification. CBDA reduced carrageenan-induced inflammatory hyperalgesia and, at otherwise sub-effective doses, cooperated pharmacodynamically with Δ^9^-THC [112]. CBC, like CBD, engages the descending antinociceptive pathway, with its antinociception sensitive to TRPA1 and adenosine A_1_ blockade [105], whereas direct analgesic evidence for CBN remains minimal; CBG’s analgesic effects appear noradrenergically mediated, consistent with its α_2_-adrenoceptor agonism [42]. The neuropathic pain dataset is notably sparse: the only clinical trial, in which CBDV (400 mg·day^−1^ for four weeks) produced numerically worse outcomes than placebo in HIV-associated neuropathic pain [126], is directionally unfavorable. The literature does not support broad analgesic claims for minor cannabinoids; current evidence is restricted to acute inflammatory settings.

In substance use disorders (SUD), Δ^8^-THCV has the strongest preclinical signal. Xi et al. [123] demonstrated anti-nicotine effects across self-administration, cue-conditioned relapse, conditioned place preference, and withdrawal phenotypes, with dose-dependence established for self-administration and relapse. These results are consistent with CB_1_ antagonism and CB_2_ agonism in reward circuitry, though not formally shown to account for the full behavioral profile. Peters et al. [132] reported satisfactory tolerability in a Phase I study, supporting progression to efficacy testing. In the opioid domain, historical data suggesting cannabinoid attenuation of morphine withdrawal (CBN, Δ^8^-THC) predate modern analytical methods and are hypothesis-generating at best. CBDA was ineffective against cocaine-seeking, consistent with serotonergic modulation being insufficient for dopaminergic reinforcement [113]. Δ^8^-THCV currently has the most compelling preclinical anti-addiction profile in this class, but it rests largely on a single laboratory program and requires independent replication.

Summary: The best evidence is Δ^9^-THCV and CBDA in acute inflammatory pain (antagonist-sensitive) and Δ^8^-THCV in nicotine addiction (effects across multiple paradigms, dose-related for self-administration and relapse, with Phase I tolerability), but both domains are dominated by single-laboratory findings in narrow paradigms, with neuropathic pain effectively untested and its sole trial directionally unfavorable. Current evidence does not support broad analgesic claims and limits realistic development to acute inflammatory pain, with Δ^8^-THCV as the leading anti-addiction candidate pending independent replication. Against Box 1, no instance in this domain meets criterion 1: the sensitivity of Δ^9^-THCV to both CB_1_ and CB_2_ antagonists in inflammatory pain reflects concurrent engagement of two receptor systems rather than a reversal in which one displaces the other, and no analgesic or anti-addiction effect reviewed here has been shown to change sign with dose, state, or model.

## 6. Human and Early-Phase Clinical Evidence

The preceding sections established substantial in vitro and preclinical evidence positioning several minor cannabinoids as neurotherapeutic candidates. The translational gap from rodent models to human therapeutics is, however, considerable: an umbrella review of 122 systematic reviews spanning 54 human diseases reported that only ~5% of animal-tested interventions ultimately receive regulatory approval [133], although this aggregate figure may not precisely reflect translation rates for CNS indications. Human data are presently confined to CBDV, Δ^9^-THCV, CBG, CBN, and Phase I safety profiling of Δ^8^-THCV; the CBG evidence derives from a single placebo-controlled crossover trial reporting acute reductions in anxiety and stress in healthy adults [134]. This section evaluates that evidence, with particular attention to negative and null findings that calibrate translational expectations. Table 6 provides a structured summary of all human clinical evidence for minor cannabinoids with CNS relevance.

### 6.1. CBDV Clinical Trials

Epilepsy: The most advanced clinical program for any minor cannabinoid is GW Pharmaceuticals’ development of CBDV (GWP42006). A Phase IIa randomized, double-blind, placebo-controlled trial (NCT02369471/NCT02365610) enrolled 162 adults with inadequately controlled focal seizures [135]. Participants were titrated to 800 mg CBDV twice daily (1600 mg·day^−1^), maintained at this dose for six weeks, and tapered over twelve days. CBDV reduced focal seizure frequency by 40.5%, while the placebo arm showed a 38% reduction, with no significant difference between groups on the primary or secondary endpoints. Under the framework developed in Section 7, this null result is most parsimoniously read as evidence that the tested dose, formulation, and population were insufficient to show efficacy, rather than definitive evidence against CBDV’s anticonvulsant pharmacology, which retains preclinical support across multiple paradigms (Section 5.1). Contributing factors include the high placebo response typical of cannabinoid trials, exposure limitations relative to effective preclinical doses (the clinical dose was approximately 8- to 10-fold lower on a mg·kg^−1^ basis), and probable loss of entourage-dependent efficacy with a purified isolate. CBDV was generally well tolerated; the safety laboratory data are detailed in Section 6.5.

Autism spectrum disorder: Two double-blind, placebo-controlled, crossover neuroimaging studies provide the first human pharmacodynamic evidence for a minor cannabinoid in a neurodevelopmental context. Pretzsch et al. [128] used magnetic resonance spectroscopy (MRS) in 34 participants to show that a single 600 mg oral dose of CBDV increased basal ganglia glutamate; the increase correlated negatively with baseline glutamate in the ASD group only, consistent with homeostatic normalization of excitatory tone. A companion study in 28 participants found that CBDV shifted atypical striatal functional connectivity toward neurotypical profiles [129]. The same group applied an equivalent single-dose crossover design to the parent compound CBD, which modulated resting-state low-frequency activity and functional connectivity in ASD [139], indicating that acute neuroimaging is sensitive to cannabinoid action in this population. These findings constitute proof of mechanism, not proof of efficacy: symptomatic outcomes were not assessed, and results from a registered pediatric efficacy trial have not been reported. A separate Phase I trial of CBDV (10 mg·kg^−1^·day^−1^) in five girls with Rett syndrome confirmed pediatric tolerability [130].

Neuropathic pain: CBDV (400 mg·day^−1^ for four weeks) produced numerically higher pain intensity than placebo in HIV-associated neuropathic pain [126]. This directionally unfavorable result is discussed below.

### 6.2. Δ^9^-THCV Exploratory Studies

Δ^9^-THCV is mechanistically promising for psychosis based on its CB_1_ antagonism combined with 5-HT_1_A signaling enhancement, but it is not yet clinically validated for this indication. The current human evidence consists of small Δ^9^-THC challenge or neuroimaging studies and safety data derived largely from non-psychiatric populations.

Attenuation of Δ^9^-THC effects: Englund et al. [136] administered 10 mg oral Δ^9^-THCV daily for five days to ten healthy male cannabis users in a placebo-controlled crossover design, followed by intravenous Δ^9^-THC challenge (1 mg). Δ^9^-THCV attenuated subjective intoxication in nine of ten volunteers and dampened both delayed verbal recall impairment and heart rate elevation. Memory intrusions increased, psychotic-like symptoms on the Community Assessment of Psychic Experiences were unchanged, and working memory showed only preliminary improvement [3]. The study was underpowered (*n* = 10) and conservatively dosed, precluding definitive conclusions about the efficacy of CB_1_ antagonism in humans.

Neural reward modulation: Two single-dose functional magnetic resonance imaging (fMRI) studies in healthy volunteers (*n* = 20) reported altered activation of reward and salience circuits with Δ^9^-THCV [137] and shifted amygdala–prefrontal connectivity in the opposite direction to that observed in obesity [140]. Both are mechanistically suggestive but underpowered to draw inferences about efficacy.

Type 2 diabetes: The most substantial Δ^9^-THCV trial to date is a randomized, double-blind, placebo-controlled, parallel-group pilot in 62 non-insulin-treated patients with type 2 diabetes [53]. Over 13 weeks, participants received Δ^9^-THCV (10 mg·day^−1^), CBD (200 mg·day^−1^), two CBD:Δ^9^-THCV combinations, or placebo. The primary high-density lipoprotein-cholesterol endpoint was not met, although Δ^9^-THCV monotherapy improved several secondary glycemic endpoints compared with placebo (Table 6). Two features carry over to the CNS context: no body-weight change occurred, distinguishing Δ^9^-THCV pharmacologically from rimonabant; and co-administration with CBD abolished Δ^9^-THCV’s benefits, demonstrating that cannabinoid–cannabinoid interactions can be antagonistic as well as synergistic, with direct implications for combination formulation design. For the psychosis rationale, the trial’s principal value is its 13-week safety record at 10 mg·day^−1^ in a non-psychiatric population rather than its metabolic efficacy.

Safety profiling: Phase I studies of Δ^8^-THCV in healthy volunteers reported no serious adverse effects across dose-ranging protocols (12.5–50 mg oral) [132], providing a class-level safety foundation that supports advancement to efficacy trials.

### 6.3. CBN: Sleep and Sedation Claims

CBN is widely marketed as “the sleepy cannabinoid,” a reputation reflecting its accumulation in aged cannabis via oxidative degradation of Δ^9^-THC [141]. The earliest clinical study enrolled five male volunteers who received oral CBN (50 mg), Δ^9^-THC (12.5 mg), or combinations [142]. The combination modestly increased subjective drowsiness compared with Δ^9^-THC alone, whereas CBN administered alone produced no significant effects on any physiological or psychometric measure. Preclinical data had until recently been equally thin: one mouse study reported an increase in barbiturate-induced sleep time [143], whereas another did not [144]. The first objective preclinical characterization came only in 2025, when Arnold et al. (2025) [145] used wireless polysomnographic telemetry in rats to show that CBN produced biphasic but ultimately marked increases in both non-rapid eye movement (NREM) and rapid eye movement (REM) sleep, the NREM effect comparable to zolpidem; the active metabolite 11-OH-CBN was a meaningful contributor and showed substantially stronger CB_1_ engagement than CBN itself [145]. CBN’s intrinsically weak CB_1_ affinity (Kᵢ ≈ 211 nM, determined by [^3^H]CP-55940 displacement in rat brain synaptosomal membranes, with values across studies ranging from approximately 120 to 320 nM depending on species and assay conditions) cannot account for these effects at typical exposures; a metabolite-driven mechanism is therefore more parsimonious and reframes the assumption that CBN is itself the active sedative [43]. Recent clinical evidence is more supportive than the 1975 study, though it does not yet settle the question: in a large, decentralized, placebo- and melatonin-controlled trial, purified CBN at 25 to 100 mg improved self-reported sleep quality to a degree comparable to 4 mg melatonin [138]. These effective doses lie well above the sub-5 mg concentrations at which most commercial CBN products are sold, which are almost certainly below the threshold for either the parent compound or sufficient metabolite generation. As the trial relied on subjective self-reports rather than objective sleep measurements, controlled human trials incorporating polysomnography remain required to confirm the signal and translate it into clinical practice [141].

### 6.4. Negative, Null, and Inconclusive Findings

The clinical dataset, though limited, reveals a consistent pattern when examined through a translational lens. Where adequately powered efficacy trials exist, results have been negative (CBDV in epilepsy [135] and neuropathic pain [126]) or mixed (Δ^9^-THCV in diabetes: primary endpoint missed, secondary endpoints improved [53]). The Δ^9^-THCV challenge study [136], often cited as a psychosis-relevant pilot, is best characterized as inconclusive due to its limited power and conservative dosing. CBDV’s ASD neuroimaging data demonstrate pharmacodynamic engagement without symptomatic improvement [128,146]. CBN’s sleep claims remain effectively untested in humans, lacking any adequately designed trials. The most instructive theme across these results is a systematic mismatch, in dose, formulation, and combination strategy, between the pharmacological conditions under which preclinical efficacy was established and those tested clinically. Section 7.5 develops this disconnect mechanistically.

### 6.5. Safety and Tolerability

Across available studies, minor cannabinoids show a generally favorable safety profile. CBDV at 1600 mg·day^−1^ over eight weeks of total exposure (titration plus six-week maintenance) in adults with focal epilepsy was generally well tolerated, with treatment-emergent adverse events reported in 72.8% of CBDV-treated participants versus 48.1% of placebo participants; the three most common events were diarrhea, nausea, and somnolence, and the incidence of serious adverse events was low (3.7% vs. 1.2%) [135]. Three CBDV-treated and one placebo participant developed serum alanine or aspartate aminotransferase elevations exceeding three times the upper limit of normal; two CBDV participants discontinued for this reason, but no cases met the potential Hy’s Law criteria [135]. These modest hepatic findings were not characterized further, for example, by stratification for concomitant valproate use, the principal driver of transaminase elevations in CBD trials. CBDV’s parent-compound hepatotoxicity therefore remains incompletely characterized, and pre-specified hepatic monitoring should be expected for all Phase II minor cannabinoid trials, particularly those exceeding 600 mg·day^−1^ chronic exposure. No adverse reactions were reported at 400 mg·day^−1^ over four weeks in HIV-associated neuropathic pain [126], and satisfactory pediatric tolerability was observed at 10 mg·kg^−1^·day^−1^ in Rett syndrome [130].

Δ^9^-THCV showed no serious adverse effects in the Δ^9^-THC-challenge study (10 mg·day^−1^ for five days) [136], or the 13-week diabetes trial (10 mg·day^−1^) [53], supported by Δ^8^-THCV Phase I data [132]. Human CBN safety data, long limited to a 1975 five-volunteer study reporting no adverse effects at doses up to 50 mg [142], now include a large placebo-controlled trial of purified CBN (25–100 mg) with a tolerability profile comparable to melatonin [138].

The absence of psychotropic effects, consistent with the negligible CB_1_ agonist efficacy established earlier, constitutes a defining safety advantage for this compound class. Outstanding considerations include the lack of long-term safety data beyond 13 weeks and the lack of evaluation in special populations (elderly, pregnant, and hepatic- or renal-impaired individuals).

Taken together, the studies reviewed above place the eight focal compounds across a wide range of clinical maturity. CBDV is the most advanced: it has completed a Phase IIa epilepsy trial and an HIV-associated neuropathic pain trial, both of which failed to separate from placebo [126,135], and a single-dose neuroimaging study in autistic adults [139], while a randomized, placebo-controlled Phase II trial of CBDV for irritability in autistic children is ongoing (ClinicalTrials.gov NCT03202303). Δ^9^-THCV has completed several early-phase studies, including a randomized controlled trial in type 2 diabetes with improvements in secondary glycemic endpoints [53], a Δ^9^-THC Challenge psychosis pilot [136], and a small placebo-controlled trial of a THCV–CBD combination reporting weight loss and metabolic improvement [147]. CBN has been evaluated for sleep in a large placebo- and melatonin-controlled trial of a purified isolate, which improved self-reported sleep quality across 25–100 mg doses [138], though objective polysomnographic confirmation is still awaited. The remaining focal compounds (CBG, CBC, CBDA, CBGA, and Δ^9^-THCA), together with the semi-synthetic derivatives (VCE-003, VCE-003.2, CBDA-ME), have no controlled human efficacy data and remain at the preclinical or observational stage. This distribution, in which mechanistic breadth far outstrips clinical maturity, defines the translational problems the following section addresses: the pharmacokinetic, drug–drug interaction, and formulation barriers that constrain these compounds, and the biomarker-guided framework needed to prioritize which compound–indication pairs to advance.

## 7. Pharmacokinetics, Translational Barriers, and Precision Development

### 7.1. Pharmacokinetics and CNS Exposure

All neutral phytocannabinoids share a terpenophenolic scaffold that confers high lipophilicity but poor aqueous solubility. For the extensively characterized cannabinoids Δ^9^-THC and CBD, and for the structurally analogous CBDV, this physicochemical profile produces low oral bioavailability (on the order of 5–10%) and extensive first-pass hepatic metabolism [2,8]. Directly measured absolute bioavailability data for CBG, CBN, and Δ^9^-THCV in humans remain sparse; the limited human pharmacokinetic data available (e.g., for CBG; [148]) are nonetheless consistent with a similarly low-exposure, fat-sensitive absorption profile. For CBDV, allylic oxidation at C-6 and C-7 constitutes the principal metabolic pathway, although human metabolite identification remains incomplete and the pharmacological activity of CBDV metabolites has not been characterized [2]. This gap is potentially consequential: CBD undergoes extensive hepatic oxidation to 7-hydroxy-CBD and then 7-carboxy-CBD, and whereas 7-hydroxy-CBD is itself pharmacologically active, the independent activity of the terminal 7-carboxy-CBD metabolite is less well characterized [149]. By analogy, CBDV metabolites may contribute meaningfully to clinical effects and warrant systematic investigation.

Distribution into the CNS is governed principally by passive transcellular diffusion across the blood–brain barrier [4]. Neutral cannabinoids (CBDV, CBG, CBN, and Δ^9^-THCV) generally achieve higher brain-to-plasma ratios than their acidic precursors, because decarboxylation removes the polar carboxylate group that impedes membrane transit. The acidic cannabinoids CBDA, Δ^9^-THCA, and CBGA therefore exhibit limited CNS access, with brain-to-plasma ratios below 0.04, rapid absorption (tₘₐₓ 15–45 min), and half-lives under 4 h [40].

These determinants are reflected in the principal systematic comparative pharmacokinetic study to date on several minor cannabinoids [150], which revealed pronounced interspecies differences. CBDV achieved higher plasma and brain concentrations in rats than in mice. Δ^9^-THCV demonstrated extensive brain penetration exceeding plasma levels but was rapidly eliminated after oral dosing (t_1/2_ < 1.5 h versus > 5 h after intraperitoneal administration). CBG exhibited similar plasma profiles across species but a dissociation between plasma exposure and brain penetration in rats. Collectively, these findings render plasma pharmacokinetics an unreliable surrogate for CNS exposure and complicate human dose extrapolation from rodent data.

### 7.2. Formulation, Prodrug, and Combination Strategies

The limited CNS access of acidic cannabinoids is at least partially formulation dependent. Anderson et al. [40] demonstrated that CBDA brain penetration increased markedly in a Tween-based formulation relative to an oily vehicle, an effect not observed for Δ^9^-THCA. The compound-specificity of this enhancement argues against a nonspecific micellar mechanism and underscores the need to co-optimize the delivery vehicle alongside receptor pharmacology. Transdermal delivery offers a complementary route that bypasses first-pass metabolism while achieving high local tissue concentrations; Hammell et al. [151] established this principle for CBD in a rat arthritis model, although it remains untested whether minor cannabinoids achieve comparable transdermal flux.

Prodrugs and semi-synthetic derivatives address the bioavailability problem through molecular design. VCE-003 and VCE-003.2, engineered for PPARγ selectivity with minimal CB_1_/CB_2_ engagement, show consistent efficacy across neurodegenerative models (Section 5.2); however, the effective intraperitoneal dose (10 mg·kg^−1^) had to be doubled when administered orally [48], indicating that bioavailability remains limiting even for optimized derivatives. CBDA-ME (HU-580) exemplifies the stabilized-analog strategy: unlike CBDA, it resists thermal decarboxylation, retains potent 5-HT_1_A allosteric potentiation, and reproduces CBDA’s anxiolytic profile at doses as low as 0.01 µg·kg^−1^ [47]. Notably, effective antidepressant doses of CBDA-ME are substantially higher and sex-dependent: 1 mg·kg^−1^ in males versus 5–10 mg·kg^−1^ in females in the forced swim test [90,91], compared with an anxiolytic threshold near 0.01 µg·kg^−1^ [47]. This span of approximately five orders of magnitude likely reflects not merely differences in assay sensitivity but a shift in the dominant pharmacological mechanism (Section 4.4): the anxiolytic and antiemetic endpoints are mediated by 5-HT_1_A allosteric potentiation, which has an intrinsic efficacy ceiling and amplifies ambient serotonin most effectively when serotonergic tone is already elevated by stress or an emetic challenge, whereas antidepressant-like endpoints may require additional targets or higher receptor occupancy beyond that allosteric ceiling. The accompanying sex-dependent dosing differential reinforces the need for sex-stratified clinical dose-finding (Section 7.5).

Multi-component formulations introduce a further dimension. The “entourage effect” hypothesis proposes that phytocannabinoids and terpenes act synergistically in whole-plant preparations [152], but evidence specific to minor cannabinoids reveals both synergistic and antagonistic interactions. In preclinical seizure models, CBDV-rich botanical extracts outperformed the purified isolate, suggesting synergy, whereas the antagonistic CBD–Δ^9^-THCV interaction in the diabetes trial [53] points in the opposite direction, likely reflecting competitive CYP metabolism or opposing receptor-level effects. Cannabinoid–cannabinoid interactions are thus significant but not uniformly beneficial, and the design of combination formulations requires empirical optimization rather than a presumption of synergy.

### 7.3. Drug–Drug Interaction Risks

CYP-mediated metabolism of minor cannabinoids creates clinically relevant potential for drug–drug interactions, particularly in polypharmacy-intensive indications such as epilepsy and schizophrenia. CBD strongly inhibits CYP3A4 and CYP2C19 and has demonstrated dose-dependent hepatotoxicity signals in clinical use [8,9]. CBDV and Δ^9^-THCV also inhibit CYP2D6, a highly polymorphic enzyme involved in the metabolism of numerous antidepressants, antipsychotics, and the endocannabinoid anandamide (AEA), with inhibitory affinity varying across genetic variants, although their potency is consistently lower than that of Δ^9^-THC [153]. Beyond CYP-mediated interactions, several cannabinoids, including CBN, inhibit hepatic and renal UDP-glucuronosyltransferase (UGT) enzymes at pharmacologically relevant concentrations [154], identifying phase II metabolism as an additional and largely unexplored pathway for potential drug–drug interactions. The clinical significance of these interactions has not yet been established and represents an important area for future translational and clinical investigation.

### 7.4. Preclinical–Clinical Disconnects and Biomarker-Guided Target Engagement

Three categories of disconnect explain why robust rodent data have not yet been translated into positive clinical outcomes for minor cannabinoids. The first is pharmacokinetics: the CBDV epilepsy program exemplifies this gap, with a clinical dose approximately 8- to 10-fold lower than the preclinical effective dose, compounded by the interspecies differences in brain penetration described above. The second is methodological: sample sizes were typically small (*n* = 4–8 per group), blinding was reported in only 52% of studies, and most neuroprotection studies employed acute chemical-lesion models rather than genetic models that recapitulate progressive human disease [3,4].

The third is pharmacological and centers on formulation: preclinical CBDV efficacy relied on enriched extracts rather than the purified isolate used clinically. The high placebo response characteristic of cannabinoid trials further argues for active-comparator designs (e.g., CBD or low-dose standard-of-care) and enhanced blinding. The polypharmacological nature of these agents compounds the difficulty of attributing target engagement. When a single compound simultaneously engages CB_1_, CB_2_, PPARγ, TRP channels, and 5-HT_1_A, isolating the mechanism responsible for a clinical effect is intractable without pharmacodynamic biomarkers validated as markers of target engagement for these compounds, none of which yet exist in humans [4]. Existing neuroimaging and fluid biomarker modalities, however, can be repurposed. For CBDV, proton magnetic resonance spectroscopy (^1^H-MRS) glutamate quantification and resting-state functional connectivity have already demonstrated sensitivity in ASD and require only correlation with symptomatic endpoints to serve as interim efficacy biomarkers. For Δ^9^-THCV, CB_1_ receptor occupancy PET using ^18^F-labeled radioligands such as [^18^F]MK-9470 or [^18^F]FMPEP-d_2_ [155] would define the dose–occupancy relationship needed to navigate the antagonist-to-partial-agonist switch, and no newer CB_1_ tracer has yet superseded these for human dosimetry. One caveat warrants attention: if Δ^9^-THCV behaves as a neutral antagonist rather than an inverse agonist (unconfirmed, pending a constitutive-activity assay; Section 4.1), then because the validated CB_1_ PET radioligands are themselves inverse agonists, tracer-displacement kinetics may differ from standard agonist-displacement paradigms, and dose–occupancy modeling must accommodate this asymmetry. For CBDA-ME, stress-primed amygdala reactivity fMRI would directly test the stress-conditionality hypothesis [156]. For VCE-003.2, cerebrospinal fluid (CSF) neuroinflammatory cytokine panels and translocator protein (TSPO) PET for microglial activation, ideally using a third-generation tracer such as [^18^F]BS224 to limit the influence of the rs6971 polymorphism that confounds second-generation ligands such as [^18^F]DPA-714, could serve as pharmacodynamic endpoints [70,121]. Table 7 consolidates these modalities into a staged development roadmap.

The biomarkers proposed for compound–indication stratification (Table 7) differ markedly in readiness, and it is important to distinguish the maturity of each measurement modality from its validation as a predictive stratifier of drug response. There are mature, human-ready modalities. Cannabinoid CB_1_ receptor occupancy measured by positron emission tomography using validated tracers ([^18^F]MK-9470, [^18^F]FMPEP-d_2_) directly indexes the occupancy variable central to Δ^9^-THCV’s proposed dose-dependent switch; CYP2D6 and CYP2C19 pharmacogenomic genotyping is already clinically implemented under established dosing guidance; and TSPO PET is a validated microglial-activation readout, subject to the rs6971 binding-affinity genotype. A second group is exploratory: ^1^H-MRS striatal glutamate and resting-state functional connectivity have direct proof-of-mechanism data for CBDV in autistic adults, and task-based fMRI paradigms (reward and aversion processing, threat-related amygdala reactivity, addiction cue-reactivity) together with CSF neuroinflammatory cytokine panels are well validated in their respective indications but have not been tested as response markers for these compounds. One is presently hypothetical: cerebrospinal fluid or plasma exposure as a stratifier of CBDV anticonvulsant response has not been assessed and is proposed here to bridge the preclinical-to-clinical dose gap. This tiering refers to the modalities; the predictive validity of every marker as a stratification of minor-cannabinoid response remains to be established prospectively, and the framework is accordingly presented as a testable development roadmap rather than as a set of validated companion diagnostics.

### 7.5. Patient Stratification and Precision Trial Design

Traditional undifferentiated trial designs are unlikely to succeed with minor cannabinoids because the three sources of inter-individual variability require stratification. Sex-dependent pharmacology is the most empirically established: the CBDA-ME dose differential [90,91], if recapitulated in humans, would mandate sex-stratified dosing, a particularly important consideration given the sex-linked prevalence of depression, anxiety, and chronic pain [epidemiology reference]. Genetic variability in drug-metabolizing enzymes is a second axis: CYP2D6 binding affinity for CBDV and Δ^9^-THCV varies across four polymorphic variants [153], and the narrow therapeutic window implied by the CBDV trial data [135] supports pharmacogenomic stratification during dose-finding. Phenotype-guided compound selection provides a third dimension, pairing each candidate with the indication its dominant mechanism best serves: CBDA/CBDA-ME for anxiety via 5-HT_1_A allosteric potentiation; Δ^9^-THCV for psychosis via CB_1_ antagonism combined with 5-HT_1_A potentiation; CBDV for neurodevelopmental conditions via CB_2_ and putative GABA_A_ engagement; and CBG derivatives for neurodegeneration via PPARγ.

These requirements translate into concrete trial-design principles. Future studies should mandate sex-balanced enrollment, pre-specified sex-stratified analyses, and should incorporate CYP2D6/CYP2C19 pharmacogenomic stratification into dose-finding protocols. Adaptive platform designs should evaluate purified isolates, defined phytocannabinoid combinations, and standardized extracts as distinct experimental arms, since entourage interactions are not uniformly synergistic [53]. The compound-specific biomarker roadmap proposed above and consolidated in Table 7 should be embedded as pre-specified interim endpoints, enabling adaptive dose selection guided by target engagement rather than clinical outcomes alone. In parallel, the outstanding safety gaps identified in Section 6.5, including the absence of long-term data, limited pediatric experience, and unevaluated special populations, must be resolved alongside the demonstration of efficacy.

Beyond the trial setting, implementation in routine care would be staged rather than immediate and tiered according to the accessibility of each stratification tool. In the near term, the framework’s clinically deployable elements require no specialized imaging: compound–indication matching, sex-stratified dosing, avoidance of antagonistic combinations such as CBD with Δ^9^-THCV, and CYP2D6/CYP2C19 genotype-guided dose adjustment are all feasible with tools already used in practice and could be embedded in prescribing guidance and electronic clinical-decision support once a compound is approved for a given indication. The research-grade biomarkers that anchor the roadmap (CB_1_ receptor occupancy PET, TSPO PET, and magnetic resonance spectroscopy) would remain confined to trials and specialist centers, where they establish the compound–response relationship and, for difficult cases, confirm target engagement. For CB_2_-dependent candidates, this confirmation is a prerequisite rather than a refinement, given the poor rodent-to-human concordance in CB_2_ pharmacology (Section 4.2); these modalities are not proposed for routine, universal use. The intended trajectory is that once a biomarker–response link is prospectively validated in the trials proposed above, it will be translated into an accessible surrogate (a blood-based marker, a validated symptom scale, or a digital or wearable measure, such as actigraphy, for CBN-associated sleep endpoints) suitable for general clinical use. In practice, a clinician would then match compound to indication and to patient subgroup, initiate dosing within the therapeutic window identified for that pairing, titrate cautiously to avoid crossing the occupancy or state boundaries at which the net effect can reverse, and reserve target-engagement confirmation for non-responders before dose escalation or a change of agent. This pathway is contingent on regulatory approval and prospective biomarker validation; the framework is, for now, a development roadmap whose routine-care elements would enter practice incrementally as each is validated.

## 8. Future Directions and Conclusions

The preceding sections established the pharmacological profiles, preclinical evidence, clinical dataset, and translational barriers facing eight minor phytocannabinoids. Three questions remain: which compounds merit advancement to proof-of-concept trials, what regulatory frameworks govern their development, and what conclusions can be drawn from the current evidence base. This section addresses each in turn, proposing a tiered compound prioritization grounded in the convergence criteria applied throughout this review, surveying the evolving regulatory landscape in the United States and European Union, acknowledging the limitations of narrative synthesis, and distilling the review’s central conclusions.

### 8.1. Tiered Prioritization of Compounds for Clinical Development

**Figure 4:**
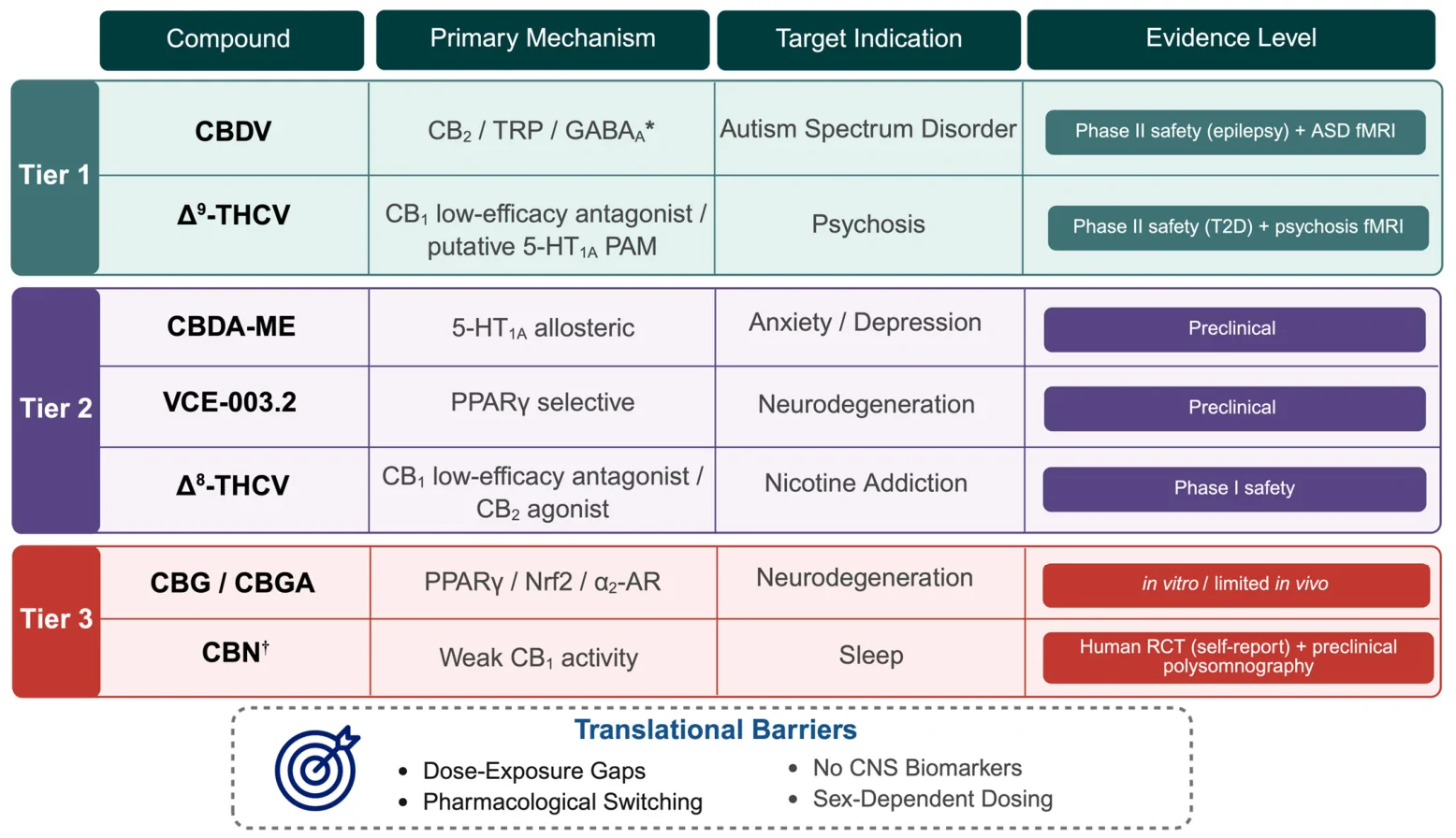
Translational evidence pipeline and tiered prioritization of minor phytocannabinoids. Each row lists a compound, its primary mechanism, target indication, and highest level of translational evidence; band color denotes the tier (teal, Tier 1; purple, Tier 2; coral, Tier 3). Asterisk (*), putative mechanism; dagger (†), tier-boundary compound. Tier 1 (strongest preclinical and early-human support): CBDV, acting at CB_2_ and TRP channels with putative GABA_A modulation, for autism spectrum disorder, supported by neuroimaging and Phase II safety data from epilepsy; Δ^9^-THCV, combining low-efficacy CB_1_ antagonism with 5-HT_1_A enhancement, for psychosis, supported by challenge fMRI and Phase II safety data from type 2 diabetes. Tier 2 (preclinical, strong rationale): CBDA-ME (5-HT_1_A positive allosteric modulator; anxiety and depression), VCE-003.2 (PPARγ-selective; neurodegeneration), and Δ^8^-THCV (CB_1_ antagonist and CB_2_ agonist; nicotine dependence, Phase I). Tier 3 (limited or stage-specific): CBG and CBGA, acting at PPARγ, Nrf2, and α_2_-adrenergic receptors, for neurodegeneration; and CBN, with weak CB_1_ activity and a large human trial of self-reported sleep outcomes alongside preclinical polysomnography via its 11-hydroxy-CBN metabolite, at the Tier 2/3 boundary. The dashed box summarizes cross-cutting translational barriers. Created in BioRender. Bagher, A. (2026) https://BioRender.com/wbhk0x9.

The evidence synthesized across the preceding sections permits a tiered prioritization based on the convergence of preclinical efficacy, mechanistic clarity, human safety data, and pharmacokinetic tractability (Figure 4). The prioritization is evidence-informed rather than efficacy-validated: no minor cannabinoid currently has randomized controlled trial evidence of symptomatic clinical benefit in its proposed indication. The tiers, therefore, identify the candidates for which proof-of-concept testing is best supported by available evidence, not candidates for which clinical utility has been demonstrated.

Tier 1: Rational candidates for early proof-of-concept clinical trials. Tier 1 compounds are not efficacy-validated therapeutics. They are the most rational candidates for early proof-of-concept testing, combining mechanistic plausibility, convergent preclinical signals across multiple disease models, preliminary human target-engagement or safety evidence, and tractable biomarker-guided trial designs. CBDV for autism spectrum disorder is supported by preclinical behavioral efficacy in a valproate ASD model [117], with convergent benefit in MeCP2-mutant Rett syndrome mice [116,146], each from a single laboratory; human proof-of-mechanism neuroimaging demonstrating pharmacodynamic target engagement [128,129]; established safety at 1600 mg·day^−1^ in adults [135]; and a registered pediatric trial. Two caveats apply. No symptomatic efficacy data yet exist for CBDV in ASD: the neuroimaging studies demonstrate glutamatergic and connectivity modulation but not behavioral improvement. The available safety data, moreover, derive from the epilepsy indication rather than the ASD population. The convergence of preclinical behavioral efficacy, human pharmacodynamic engagement, and a favorable safety profile nonetheless positions CBDV as the most advanced minor cannabinoid for proof-of-concept testing. Its CB_2_/TRP-mediated mechanism, with putative GABA_A_ involvement, is orthogonal to risperidone and aripiprazole, the only U.S. Food and Drug Administration (FDA)-approved agents for ASD-associated irritability, both of which carry substantial metabolic liabilities that are particularly concerning in pediatric populations [157].

Δ^9^-THCV for psychosis combines CB_1_ antagonism with 5-HT_1_A signaling enhancement, a mechanistic profile aligned with positive, negative, and cognitive symptom domains [3], and is supported by safety data at 10 mg·day^−1^ over periods up to 13 weeks [53,136]. The psychiatric safety record remains thin, the 13-week data deriving from a metabolic (type 2 diabetes) rather than a psychiatric population. The non-dopaminergic mechanism could, in principle, avoid the extrapyramidal, metabolic, and hematological liabilities of existing antipsychotics. This independence may not be absolute, however, since CB_1_ engagement can influence dopaminergic signaling indirectly through CB_1_–D_2_ receptor heteromerization, which is itself sensitive to concurrent antipsychotic treatment [158]. Both the proposed advantage and this caveat are testable in proof-of-concept trials.

Tier 2: Advanced preclinical candidates. CBDA-ME for anxiety and depressive disorders shows potency advantages over CBD at 5-HT_1_A allosteric potentiation, together with thermal stability that CBDA lacks [47]. Unlike selective serotonin reuptake inhibitors, which require weeks for onset and carry sexual dysfunction and emotional blunting as common dose-limiting effects, CBDA-ME’s direct allosteric potentiation produced acute-onset effects in preclinical models [87,90]. The sex-dependent dosing differential must, however, be resolved before clinical dose selection can proceed.

VCE-003.2 shows consistent neuroprotective efficacy across HD, PD, MS, and ALS models *via* PPARγ activation but lacks human data. As noted in Section 5.2, this multi-model evidence derives predominantly from a single consortium, and independent replication remains the principal outstanding requirement. Δ^8^-THCV for nicotine addiction combines robust preclinical anti-nicotine efficacy, including reduced self-administration, cue-conditioned relapse prevention, and withdrawal attenuation [123], with Phase I safety data [132].

Tier 3: Early discovery. CBG and CBGA warrant continued mechanistic investigation for neurodegenerative applications, but their consistent failure across psychiatric paradigms and the absence of dual-antagonist resolution of their competing pharmacology (5-HT_1_A antagonism combined with α_2_-adrenergic agonism, in the case of CBG) relegate them to the discovery phase [3]. CBN occupies an intermediate position. Recent objective polysomnography in rats confirmed sedative effects comparable to those of zolpidem and identified the active metabolite 11-OH-CBN as a meaningful contributor [145], providing the first credible preclinical foundation for CBN’s marketed sleep claim. However, no adequately powered controlled human trial incorporating polysomnography has been conducted, and commercial product dosing remains substantially below preclinically active concentrations. CBN therefore sits at the Tier 2/Tier 3 boundary, with focused proof-of-concept clinical trials for insomnia, ideally incorporating metabolite-aware dosing and polysomnographic endpoints, the appropriate next step.

### 8.2. Regulatory and Translational Landscape

The regulatory landscape creates both opportunities and constraints for the development of minor cannabinoids. In the United States, Section 781 of the Continuing Appropriations and Extensions Act, 2026 (P.L. 119-37, Division B) redefined hemp to incorporate total tetrahydrocannabinol content and imposed a 0.4 mg per finished-product container cap, with enforcement scheduled for 12 November 2026 [13]. Non-intoxicating minor cannabinoids such as CBDV, CBDA, and CBG can, in principle, be formulated to comply with these thresholds, positioning them favorably relative to Δ^9^-THC-class compounds.

In the European Union, the 2026 update by the European Food Safety Authority Panel on Nutrition, Novel Foods and Food Allergens to its 2022 statement on the safety of CBD as a novel food established a provisional safe intake level of 0.0275 mg·kg^−1^ body weight per day, approximately 2 mg·day^−1^ for a 70 kg adult, derived by benchmark-dose modeling with an uncertainty factor of 400 [14]. This level applies only to CBD of at least 98% purity, free of nanoparticles, produced under a process considered safe, and for which genotoxicity has been excluded. The Panel concluded that safety cannot currently be established for individuals under 25, for pregnant or lactating women, or for those on concurrent medications. Although CBD-specific and addressed to the novel-food rather than medicinal-product pathway, it signals an increasingly conservative posture toward cannabinoid exposure in vulnerable populations, which minor cannabinoids with controlled human safety data may be better positioned to navigate.

Beyond the United States and the European Union, the regulatory environment of the Gulf and the wider Middle East merits brief consideration, given the region’s distinct translational constraints and emerging research capacity. Across most Arab states, cannabis and cannabis-derived products remain prohibited, with Lebanon’s 2020 authorization of medical and industrial cannabis the principal exception; non-intoxicating cannabinoids such as CBD are generally controlled as stringently as Δ^9^-THC rather than treated as distinct entities [159]. In Saudi Arabia, medicinal products are evaluated by the Saudi Food and Drug Authority within controlled-substance frameworks, and peer-reviewed data on the regulatory handling of individual minor cannabinoids remain sparse, so compound-specific regional statements should be made cautiously. Two structural barriers are well documented for the Arab world: a scarcity of controlled clinical research and limited dedicated funding [159]. At the same time, Saudi Arabia’s Vision 2030 health-transformation agenda, including the Saudi Human Genome Program and the expansion of precision-medicine infrastructure, offers a plausible long-term platform for the pharmacogenomically stratified, biomarker-guided development advocated here, for example, CYP2D6-informed dosing of CBDV and Δ^9^-THCV [160]. Realizing this potential will nonetheless require dedicated pharmacovigilance and controlled-trial capacity that does not yet exist regionally for this class of compounds.

In pharmaceutical development, two pathways merit consideration. The FDA’s Botanical Drug Development guidance [12] permits standardized extract-based products to pursue marketing approval through the standard Investigational New Drug and New Drug Application pathway (21 Code of Federal Regulations Part 312, 21 Code of Federal Regulations Part 314), with botanical-specific provisions for chemistry, manufacturing, and controls documentation that are potentially advantageous for entourage-dependent formulations. Alternatively, isolated compounds may pursue conventional Investigational New Drug applications, which offer clearer intellectual property protection but forgo the benefits of multi-component interactions.

Further considerations shape the realistic translational pathway for this class. Industry development of minor cannabinoids has visibly contracted since 2021. Jazz Pharmaceuticals completed its acquisition of GW Pharmaceuticals in May 2021 for $7.2 billion, inheriting the GWP42006 (CBDV) program, as well as Epidiolex^®^ and nabiximols. The pivotal CBDV focal-seizure trial had already failed to meet its primary endpoint (read out in 2018; published in [135]), and no minor cannabinoid has since advanced into Phase III for a CNS indication. To date, no minor cannabinoid has received Breakthrough Therapy designation from the U.S. Food and Drug Administration (FDA) or Priority Medicines (PRIME) designation from the European Medicines Agency (EMA). PRIME is an EMA program designed to accelerate the development and regulatory assessment of medicines that address unmet medical needs. Under this scheme, academic institutions and small and medium-sized enterprises (SMEs) may apply for PRIME designation based on proof-of-principle evidence, namely compelling nonclinical data together with first-in-human tolerability, before the more extensive proof-of-concept clinical evidence generally expected from commercial sponsors [161]. The evidence class proposed here is well matched to that threshold: the pharmacodynamic engagement signatures in Table 7 are those through which an academic or consortium sponsor would most plausibly substantiate such a request. Academic and consortium-led pathways (investigator-initiated INDs in the United States, NIH-funded mechanism-of-action trials, and European Medicines Agency Scientific Advice with eventual PRIME applications for compounds meeting the unmet-need threshold) are therefore the more realistic translational route for this class. The non-intoxicating, regulatory-favorable, mechanistically distinct profiles of CBDV (autism spectrum disorder), Δ^9^-THCV (psychosis), and VCE-003.2 (neurodegeneration) suit them to such pathways, provided trial designs incorporate the biomarker and stratification principles in Section 7.

### 8.3. Limitations

Several limitations should be acknowledged. This is a narrative rather than a systematic review; the search strategy was not registered, and study selection did not follow a formalized protocol. This approach permits cross-domain mechanistic integration that PRISMA constraints make difficult, at the cost of a formalized, quantitative risk-of-bias assessment of the primary literature. Reliance on prior reviews [2,3,4,5] to help identify primary studies may also have shaped the literature visible to this synthesis, although the independent database searches were intended to mitigate this effect.

A broader interpretive limitation is that the clinical relevance proposed for several compounds rests substantially on receptor pharmacology and preclinical data that human studies have not yet tested. Δ^9^-THCV is the clearest example: it has the most fully developed receptor rationale in the class, yet its human evidence comprises only a small challenge study, two single-dose imaging studies, and a metabolic trial that missed its primary endpoint, none of which evaluated its proposed psychiatric indication. The strength of a mechanistic case should not be read as clinical validation, a caveat that applies across the compounds prioritized here. A related translational caveat applies specifically to CB_2_-mediated mechanisms: because CB_2_ is the least conserved cannabinoid receptor between rodents and humans, in both expression and ligand pharmacology (Section 4.2), the anti-neuroinflammatory conclusions drawn for the PPARγ/CB_2_/Nrf2 axis rest on rodent data whose CB_2_ component requires confirmation at the human receptor before it can be assumed to translate.

The review is limited to English-language peer-reviewed publications, and publication bias may overrepresent positive findings, particularly for compounds with commercial development interest. Human evidence is also limited by sex imbalance in early studies; the Δ^9^-THC-challenge study of Englund et al. (2016) [136], for example, enrolled only ten healthy male cannabis users, limiting generalizability and reinforcing the need for adequately powered, sex-balanced studies. The heterogeneity of the preclinical designs precluded meta-analysis, and cross-study effect-size comparisons should be interpreted with caution. The tiered prioritization proposed here is an evidence-informed framework rather than definitive clinical guidance.

### 8.4. Conclusions

Minor phytocannabinoids are a pharmacologically diverse but clinically underdeveloped class. The evidence reviewed here shows that they are not interchangeable: each possesses a unique receptor signature, shaped by sidechain length, ring-cyclization pattern, and carboxylation state, that determines its therapeutic window. Three convergent signaling axes emerge as the most robustly supported at the mechanistic level. First, the PPARγ/CB_2_/Nrf2 anti-inflammatory axis underpins neuroprotection by CBG, VCE-003.2, and Δ^9^-THCA across HD, PD, and MS models, with PPARγ activation consistently confirmed by antagonist controls; the CB_2_ contribution to this axis is less securely translatable, however, because CB_2_ is the least conserved cannabinoid receptor between rodents and humans, and its rodent-derived neuroprotective role requires confirmation at the human receptor (Section 4.2). Second, TRP channel desensitization, combined with putative GABA_A_ potentiation and 5-HT_1_A allosteric modulation, confers anticonvulsant efficacy in CBDV and CBDA, though with clear model-dependent boundaries. Third, low-efficacy CB_1_ antagonism coupled with 5-HT_1_A potentiation positions Δ^9^-THCV uniquely among these compounds for psychosis and reward-circuit disorders. Translational interpretation of the acidic members of these axes (CBDA, CBGA, and Δ^9^-THCA) is further tempered by pharmacokinetics: their poor brain penetration and, for Δ^9^-THCA, facile decarboxylation to psychoactive Δ^9^-THC (Section 2 and Section 6) mean that their CNS potential is realistically contingent on stabilized analogs or penetration-enhancing formulations rather than on the parent acids.

A central analytical contribution of this review is the pharmacological switching framework (Section 4.4): dose-, state-, or model-dependent shifts in the dominant determinant of a compound’s net effect that reverse the direction, rather than merely the magnitude, of that effect on a defined functional endpoint, complicating linear dose–response assumptions and motivating the biomarker-guided dosing developed in Section 7.

These mechanistic insights must, however, be weighed against a sobering clinical reality. Where adequately powered efficacy trials have been conducted, results have been negative (CBDV in epilepsy and neuropathic pain) or mixed (Δ^9^-THCV in diabetes). No minor cannabinoid has yet demonstrated efficacy in a randomized, adequately powered clinical trial designed to test a neurological or neuropsychiatric indication.

The path from preclinical promise to clinical validation requires resolving three interconnected translational barriers: dose–exposure uncertainty, biomarker deficiency, and formulation-dependent efficacy. The compound-specific biomarker roadmap, sex-stratified designs, pharmacogenomic stratification, and optimized formulations outlined in Section 7 provide a concrete framework for each. Progress will depend less on discovering new cannabinoids than on systematically matching existing candidates to the indications, doses, and subgroups where their distinctive pharmacology confers an advantage. The trajectory of VCE-003.2, from a promiscuous natural scaffold to a PPARγ-selective, antagonist-validated, metabolically stabilized neuroprotective candidate, offers a template for the rational optimization that much of the rest of the class still requires.

## Figures and Tables

**Table 2:** Pharmacological switching versus related concepts of context-sensitive drug action, distinguished by the reversal of the net direction of effect.

Concept	Level of Action	Net Directional Reversal?	Principal Driver	Mechanistic Basis	Illustrative Case
Pharmacological switching (this review)	System-level *net* effect	Yes, defining criterion	Dose, physiological/pathological state, or experimental model (receptor-expression context)	Shift in the dominant target, receptor population, or efficacy mode engaged	Δ^9^-THCV: CB_1_ antagonism transitioning toward partial agonism as receptor occupancy rises above threshold (candidate instance; reversal predicted, not yet demonstrated) [26,27]
Biased agonism (functional selectivity)	Single receptor	No	Ligand–receptor conformation	Preferential coupling to one transducer/pathway (e.g., G-protein vs. β-arrestin) without reversal of net direction	Ligand-dependent biased signaling at CB_1_ [83,84]
Partial agonism (low efficacy)	Single receptor	Apparent only; agonism versus functional antagonism	Endogenous agonist tone; receptor reserve	Fixed intrinsic efficacy expressed differently across systems	Δ^9^-THCV at CB_1_ (alternative account of the dose-dependent case) [85]
Protean agonism	Single receptor	Yes, but relative to constitutive activity	Constitutive receptor activity of the system	Ligand stabilizes an active state of lower efficacy than the spontaneous active state	Not demonstrated for phytocannabinoids; pertinent while Δ^9^-THCV’s neutral-antagonist status is unresolved (Section 4.1) [85,86].
Hormesis	Single measured endpoint	Yes, but dose-restricted	Dose alone	Biphasic dose–response intrinsic to a single mechanism, with opposing processes acting at different concentration thresholds on one endpoint	Biphasic (low-dose stimulatory, high-dose inhibitory) dose–response [78,79]
Context-dependent pharmacology	Variable (tissue, state, receptor reserve)	Not required	Biological or environmental context	Modulation of the magnitude or quality of effect with context; direction may or may not change	State- and tissue-dependent drug action [80]

Net directional reversal is assessed against Box 1, criterion 1. Pharmacological switching requires such a reversal as a defining feature. Biased agonism alters the downstream distribution of a response without requiring a change in its direction. Partial agonism and protean agonism can reverse the apparent direction of effect at a single receptor through system-dependent expression of a fixed intrinsic efficacy and through the constitutive activity of the preparation, respectively, but without changing which mechanism dominates. Hormesis can also reverse direction, but only as a function of dose at a single endpoint, and context-dependent pharmacology can alter the magnitude or quality of an effect, with or without a change in direction. The distinction is operationally testable. Where the reversal arises between receptor systems, antagonist sensitivity is limb-specific, each limb being abolished by a different selective antagonist, whereas a single antagonist abolishes both limbs of a hormetic response. Where the reversal arises from an efficacy-mode transition at one receptor, the two limbs differ instead in their dependence on ambient agonist tone: the low-occupancy limb is lost when endogenous tone is removed, whereas the high-occupancy limb persists.

**Table 3:** Documented instances of pharmacological switching and related context-dependent effects of minor phytocannabinoids, graded by whether they meet the directional-reversal criterion (Box 1) and by evidentiary confidence.

Compound	Context- Dependence Mode	Mechanistic Description	Translational Implication	Directional Reversal (Box 1, Criterion 1)	Confidence	Key Ref.
Δ^9^-THCV	Dose-dependent	Low-efficacy CB_1_ antagonist in vitro (IC_50_ 434 nM), with agonist-like tetrad effects in mice at 3–10 mg·kg^−1^, consistent with a transition toward partial agonism at higher exposure; antagonism and agonism were shown in separate systems and no common functional endpoint has been titrated across the transition, so criteria 1, 3, and 5 are not yet satisfied	Dose escalation may reverse therapeutic mechanism; PET-based occupancy studies needed to define the antagonist–agonist boundary	Predicted, not demonstrated	Proposed	[26,27]
CBDA	State-dependent	Anxiolytic effects emerge only under stress-primed conditions that elevate serotonergic tone required for 5-HT_1_A allosteric potentiation; inactive in unstressed animals	Clinical trials must control for baseline stress state or affective tone; unstratified populations risk false-negative results	Partial	Provisional	[87,94]
CBGA	Model-dependent	Anticonvulsant in Dravet hyperthermia paradigm and potentiates clobazam; anticonvulsant in the maximal electroshock threshold test yet proconvulsant in the 6-Hz threshold test at the same dose, strain, route and pretreatment interval; proconvulsant against spontaneous seizures at the higher dose	Anticonvulsant efficacy in one seizure paradigm cannot be assumed to generalize across seizure types; model selection determines efficacy conclusions	Yes	Proposed	[88,89]
CBDV	Model-dependent	Effective in PTZ and audiogenic seizure models but ineffective against pilocarpine-induced status epilepticus as monotherapy	Target–model alignment is critical; TRPV1 and putative GABA_A_ mechanism may be insufficient for broad excitotoxic seizure cascades	No	n/a	[54,55,95]
Δ^9^-THCV	Model-dependent	Anticonvulsant in PTZ seizure models (0.25 mg·kg^−1^) but proconvulsant in the Dravet thermal-threshold assay (3 mg·kg^−1^); the 12-fold dose difference confounds model with dose, so the reversal is not attributable to model identity under Box 1, criterion 5	Seizure model and dose must be varied independently before a model-dependent reversal can be claimed; anticonvulsant efficacy in one paradigm should not be assumed to generalize	No	n/a	[88,96]
CBDA-ME	Sex-dependent	Effective at ~1 mg·kg^−1^ in males but requires 5–10 mg·kg^−1^ in females in the forced swim test	Sex-stratified dosing mandatory in both preclinical and clinical development	No	n/a	[90,91]
Δ^9^-THCA	Analyte-dependent	Facile thermal decarboxylation to psychoactive Δ^9^-THC may alter compound identity during storage, formulation, or in vivo; effects attributed to Δ^9^-THCA via PPARγ may be confounded by CB_1_-mediated activity of the decarboxylation product	Analyte integrity must be verified throughout experiments; studies lacking decarboxylation controls are mechanistically uninterpretable	No	n/a	[4,40]
CBD + Δ^9^-THCV	Interaction-dependent	Co-administration of CBD abolishes Δ^9^-THCV’s glycemic benefits, possibly via competitive CYP metabolism or opposing receptor-level effects	Combination formulations may be counterproductive; empirical interaction testing required before co-formulation	No	n/a	[53]
CBG	Competing polypharmacology	Concurrent α_2_-AR agonism (EC_50_ 0.2 nM) and 5-HT_1_A antagonism produce opposing effects on anxiety-relevant circuits, yielding inconsistent or null anxiolytic outcomes across paradigms	Multi-target engagement does not guarantee therapeutic benefit; net pharmacological output depends on relative target engagement at a given dose	No	n/a	[3,42]

Directional reversal is assessed against Box 1, criterion 1 (a change in the sign of the net effect); partial denotes state-conditional emergence of a unidirectional effect rather than a strict sign reversal; predicted, not demonstrated denotes a reversal anticipated on mechanistic grounds but not yet observed on a common functional endpoint. Confidence tiers apply to instances that meet, partially meet, or are predicted to meet the reversal criteria: established (all Box 1 criteria met and independently replicated), provisional (criteria met but at a single laboratory), and proposed (one or more criteria not yet demonstrated). A single-laboratory finding is reported by a single research group or by groups sharing methods and reagents, without independent reproduction; a replicated finding is reproduced by a methodologically independent group or corroborated across independent paradigms. Rows scored “No” do not meet the reversal criterion and are retained as related context-dependent effects rather than switching; Δ^9^-THCA is a special case, reflecting thermal decarboxylation to Δ^9^-THC (an analytical-integrity artifact rather than a pharmacological switch).

**Table 4:** Preclinical evidence matrix for minor phytocannabinoids across CNS indications: effect direction, replication status, and mechanistic confidence.

Comp.	Epilepsy	AD	PD	HD	MS	ALS	Anxiety	Depression	Psychosis	Neurodev.	Nausea	Pain	SUD
CBG	0 ^S^	+ ^S^	-	+ ^S^	-	± ^S^	0 ^R^	-	-	-	-	+ ^S^	-
CBC	0 ^S^	-	-	-	-	± ^S^	-	+ ^R^	-	-	-	+ ^S^	-
CBN	-	-	-	-	-	± ^S^	-	-	-	-	-	+ ^S^	± ^H^
CBDA	+ ^S^	-	-	-	-	-	+ ^R^	-	0 ^S^	-	+ ^R^	+ ^S^	+ ^S^
CBGA	± ^S^	-	-	-	-	-	-	-	-	-	-	-	-
Δ^9^-THCA	0 ^S^	-	0 ^S^	+ ^S^	-	-	+ ^S^	-	-	-	-	-	-
CBDV	*±* ^R^	-	-	-	-	-	-	-	-	+ ^R^	-	0 ^S^	-
Δ^9^-THCV	± ^S^	-	+ ^S^	-	-	-	-	-	+ ^S^	-	-	+ ^S^	-
VCE-003	-	-	-	-	+ ^S^	-	-	-	-	-	-	-	-
VCE-003.2	-	-	+ ^S^	+ ^R^	-	+ ^S^	-	-	-	-	-	-	-
Δ^8^-THCV	-	-	-	-	-	-	-	-	-	-	-	-	+ ^S^

Legend: + beneficial/therapeutic signal; 0 null/negative; ± mixed or model-dependent; - no direct data identified. Superscripts indicate replication status: ^R^ independently replicated; ^S^ single-laboratory finding; ^H^ historical data predating modern methods.

**Table 5:** Mechanistic context for positive and mixed preclinical findings reported in Table 4.

Compound	Indication	Model(s)	Species; Sample Size (*n*)	Effect	Antagonist/ Genetic Validation	Mechanistic Attribution	Key Ref.
CBG	Epilepsy	PTZ (rats)	Rat *n*= 72 total; per-group *n* not stated	No seizure reduction despite confirmed Na_V_ blockade in vitro	Not performed	Na_V_ blockade alone insufficient for anticonvulsant efficacy	[56]
CBG	AD	MC65, HT22 cells	In vitro only: MC65 (human), HT22 (mouse) No in vivo *n*	Prevented amyloid-β accumulation (100 nM); oxytosis protection (EC_50_ 1.9 µM)	Partial (CB_1_/CB_2_ independence characterized)	Target not identified; protection occurs in cells reported to lack CB_1_/CB_2_ transcripts, though CB_1_ protein has been detected in HT22 cells elsewhere; no PPARγ or Nrf2 data in the cited sources	[97,98]
CBG	HD	3-nitropropionate (mice)	Mouse (C57BL/6; R6/2 transgenic) *n* = 6–8/group (5–6, huntingtin aggregates)	Reduced COX-2, iNOS, TNF-α; restored antioxidant defenses	Not performed (PPARγ inferred)	PPARγ-linked (Nrf2 restoration under LPS)	[99,100]
CBG	ALS/Neuroregeneration	Nrf2-depleted cells	In vitro only: NSC-34, RAW 264.7 (mouse) No in vivo *n*	Restored Nrf2 levels in vitro	Not performed	Nrf2-linked; not validated	[100]
CBG	Anxiety	Multiple paradigms	Mouse [27,101]; rat [102] *n* ≥ 6/group [27]; 8/group [102]; 19–20/group [101]	Consistently null anxiolytic outcomes	Not performed	Opposing α_2_-AR agonism and 5-HT_1A_ antagonism may cancel net effect	[27,101,102]
CBG	Pain	Inflammatory models	Mouse: in vivo [27]; ex vivo tissue [42] *n* ≥ 6/group [27]; 13 vas deferens, 7–20 membranes [42]	Weak antinociception in vivo; yohimbine sensitivity demonstrated in isolated vas deferens, not in the analgesia assay	Not performed	α_2_-AR agonism established ex vivo; analgesic effect not linked in vivo	[27,42]
CBC	Epilepsy	MES	Mouse (ICR albino, male) *n* = 10/group	No effect	Not performed	Not investigated	[103]
CBC	ALS/Neuroregeneration	Neural progenitors in vitro	In vitro only: adult neural stem/progenitor cells (mouse) No in vivo *n*	Increased viability; suppressed astroglial differentiation via ERK	Validated (U0126, MAPK/ERK kinase; DPCPX, adenosine A_1_)	Adenosine A_1_–ERK1/2 signalling; DPCPX blocked ERK phosphorylation and nestin up-regulation; glial fibrillary acidic protein suppression U0126-insensitive (dissociable)	[65]
CBC	Depression	Forced swim test, tail suspension test (mice)	Mouse (Swiss Webster [27]; DBA/2 [104]) *n* ≥ 6/group [27]; 7–10/group [104]	Reduced immobility in forced swim test (20 mg·kg^−1^) and tail suspension test (40–80 mg·kg^−1^)	Not performed	Unconfirmed	[27,104]
CBC	Pain	Intra-ventrolateral periaqueductal gray microinjection; tail-flick; rostral ventromedial medulla ON and OFF cell recording (anaesthetised)	Rat (Wistar, male, anaesthetised) *n* = 12–16/group; 6–8 neurons	Limited evidence; multi-target (TRPA1/CB_1_/AEA)	Validated (AM251, CB_1_; DPCPX, adenosine A_1_; AP18, TRPA1 all blocked; I-RTX, TRPV1 ineffective)	CB_1_-, adenosine A_1_- and TRPA1-dependent; TRPV1-independent. CBC elevated ventrolateral periaqueductal gray 2-AG (3.9×) and anandamide (1.7×)	[105]
CBN	ALS	SOD1-G93A (mice)	Mouse (transgenic SOD1-G93A, male) *n* = 18 total, 2 groups; per-group n not stated	Delayed symptom onset; no survival benefit	Not performed	Mechanism unresolved	[106]
CBN	Pain	Nerve growth factor-induced hypersensitivity; myofascial model	Rat (Sprague–Dawley, female) *n* = 6/group	Positive signal	Not performed	Not dissected	[107]
CBN	SUD	Opioid withdrawal (historical)	Rat (Sprague–Dawley, male) *n* = 20/group	Suggestive attenuation of morphine withdrawal	Not performed	Pre-modern methods; hypothesis-generating only	[108]
CBDA	Epilepsy	Scn1a Dravet; MES	Mouse (Scn1a^+/−^, male and female, P14–16); rat (Sprague–Dawley, male) *n* = 11–15/group (Scn1a); 6–8/dose or time point (MES)	Increased seizure threshold at 10–30 mg·kg^−1^; anticonvulsant in MES, with greater potency for the partial-spectrum extract	Not performed (inferred)	Mechanism not investigated in seizure models; 5-HT_1A_ and TRPV1 involvement inferred from other indications	[40,109]
CBDA	Anxiety	Stress-conditioned paradigms	Rat (Sprague–Dawley, male) [87]; mouse (C57BL/6J, male) [94] *n* = 6–12/group [87]; 10–17/group [94]	Anxiolytic at 0.1 µg·kg^−1^–30 mg·kg^−1^ only under stress-primed conditions; inactive in unstressed animals	Validated (WAY-100635)	5-HT_1A_ allosteric potentiation (stress-conditional)	[87,94]
CBDA	Psychosis	Methamphetamine hyperlocomotion	Rat (Sprague–Dawley, male) *n* = 12/group (within-subjects Latin square)	No attenuation	Not performed	Defines mechanistic boundary for serotonergic modulation in dopaminergic circuits	[110]
CBDA	Nausea	Conditioned gaping	Rat (Sprague–Dawley, male) *n* = 7–8/group [74]; 6–12/group [111]	~1000-fold more potent than CBD; synergy with ondansetron	Validated (WAY-100635; CB_1_-independent via SR141716A)	5-HT_1A_ allosteric potentiation	[74,111]
CBDA	Pain	Inflammatory hyperalgesia	Rat (Sprague–Dawley, male) *n* = 8/group	Positive; synergy with low-dose Δ^9^-THC	Partial (TRPV1-attributed)	TRPV1-attributed; not antagonist-tested	[112]
CBDA	SUD	Cocaine CPP	Mouse (CD1, male, postnatal day 35) *n* = 8–10/group (CPP); 6–10/group (other endpoints)	No effect	Not performed	Serotonergic modulation insufficient for dopaminergic reinforcement	[113]
CBGA	Epilepsy	Dravet hyperthermia; 6 Hz	Mouse *n* = 12–18 (hyperthermia); 12 (6 Hz)	Anticonvulsant in Dravet; potentiates clobazam; proconvulsant in 6 Hz	Not performed	Model-dependent switching (Section 4.4)	[88]
Δ^9^-THCA	Epilepsy	Dravet hyperthermia; 6 Hz; MES	Mouse *n* = 15 (hyperthermia); 11–12 (6 Hz); 12 (MES threshold)	Ineffective as monotherapy; proconvulsant in MES; anticonvulsant only with Δ^9^-THC	Not performed	Limited by poor brain penetration and decarboxylation instability	[40,114]
Δ^9^-THCA	PD	Dopaminergic neuron culture (in vitro)	In vitro only: primary mesencephalic culture, mouse (embryonic) † *n* = 3–4 independent cultures	No pro-survival effect on dopaminergic neurons (0.01–10 µM); modest increase in cell count (123% of MPP^+^-treated cultures)	Not performed	CB_1_/CB_2_ engagement unlikely given ~62- to 125-fold lower affinity; mechanism not investigated	[115]
Δ^9^-THCA	HD	3-nitropropionate (mice)	Mouse (C57BL/6, male) *n* = 9/group	Improved motor function, reduced neuroinflammation	Validated (T0070907)	PPARγ-dependent (T0070907-sensitive) except IL-6 (PPARγ-independent)	[38]
Δ^9^-THCA	Anxiety	Open-field screening	Mouse (C57BL/6, male) *n* ≥ 6/group	Modest signal; 5-HT_1A_ putative positive modulation at PPARγ-relevant concentrations	Not performed	Not fully dissected	[27]
CBDV	Epilepsy	Audiogenic DBA/2; PTZ; pilocarpine	Mouse (DBA/2; MF1); rat (Wistar Kyoto); HEK293; human CB_1_-transfected cells † *n* = 10/group (mice); 15/group (rats); in vitro *n* = 4–5	90% seizure-free in audiogenic model; ineffective vs. pilocarpine as monotherapy	Partial (CB_1_-independent)	TRPV1 desensitization proposed (in vitro); CB_1_-independent; effector mechanism not established in vivo	[54,55,95]
CBDV	Neurodevelopmental	valproic acid-exposed rats; MeCP2 Rett mice	Mouse (MeCP2-308 hemizygous male + wild-type littermates, 5 mo); rat (Sprague–Dawley, male offspring, prenatal valproic acid) *n* = 7–9/group (mouse); 5–15/group behavior, 3–5/group Western blot (rat)	Improved ASD-like behaviors; extended survival in Rett	Not performed (inferred)	Anti-inflammatory; hippocampal CB_2_ upregulation and endocannabinoid-tone restoration reported as correlates, causality not established; human MRS data supporting glutamate modulation reported separately (Section 6.1)	[116,117]
Δ^9^-THCV	Epilepsy	PTZ (rats); Dravet thermal threshold	Rat (Wistar, male, in vivo; Wistar Kyoto and outbred Berkshire, male and female, in vitro) *n* = 15–16/group (in vivo; source inconsistent); 3–5 (in vitro)	33% seizure-free at 0.25 mg·kg^−1^ (PTZ); proconvulsant at 3 mg·kg^−1^ (Dravet)	Not performed	Not dissected (no antagonist studies)	[88,96]
Δ^9^-THCV	PD	6-OHDA; LPS (mice)	Rat (Sprague–Dawley, male); mouse (CB_2_ knockout and wild-type) *n* = 5–6/group	Preserved tyrosine hydroxylase-positive nigral neurons and reduced microgliosis in both 6-OHDA rats and LPS mice	Not performed	Model-dependent: antioxidant and CB_2_-independent in 6-OHDA rats (lesion magnitude equal in knockout and wild-type; effect reproduced by CBD-enriched extract); CB_2_-attributed in LPS mice (receptor upregulated; effect mimicked by HU-308), but the compound was not tested in knockout mice	[118]
Δ^9^-THCV	Psychosis	Phencyclidine model (rats)	Rat (Sprague–Dawley, male; brainstem membranes); human 5-HT_1_A-transfected cells † *n* = 6/group (in vivo); 4–11 (in vitro)	Positive across positive, negative, and cognitive domains	Not performed	Attributed to 5-HT_1A_; antagonist validation outstanding	[3,46]
Δ^9^-THCV	Pain	Carrageenan; formalin	Mouse (C57BL/6J, male; wild-type and CB_2_ knockout); human CB_1_- and CB_2_-transfected cells † *n* = 5–10/group (in vivo); 4–8 (in vitro)	Reduced pain behaviors	Validated (CB_1_ + CB_2_ antagonists)	Blocked by both CB_1_ and CB_2_ antagonists (context-dependent)	[119]
VCE-003	MS	EAE; TMEV	Mouse (C57BL/6, female, EAE [64]; SJL/J, sex not stated, TMEV [120]); human, rat and mouse primary cells and cell lines † *n* = 6–11/group [64]; 9–12/group [120]; in vitro *n* = 3–4	Reduced severity, demyelination, axonal injury	Validated (AM630 + GW9662)	Blocked by both AM630 (CB_2_) and GW9662 (PPARγ)	[64,120]
VCE-003.2	PD	LPS-lesioned mice	Mouse (C57BL/6, male); mouse, rat and human cell lines † *n* = 4–6/group; in vitro *n* = 3–7	Reduced nigrostriatal damage	Partial (T0070907 in vivo; antagonist-insensitive in vitro)	PPARγ via canonical and alternative binding sites; T0070907-insensitive component in vitro	[121]
VCE-003.2	HD	3-nitropropionate; mutant huntingtin	Mouse (CD1 and C57BL/6, male [69]; C57BL/6N, male [122]); rat (Sprague–Dawley, male, pharmacokinetics [122]); mouse and human cell lines † *n* = 3–8/group [69]; 3–11/group [122]; in vitro *n* = 3	Improved neuronal and progenitor survival, motor performance, and subventricular zone neurogenesis	Validated (T0070907, 3-NP; GW9662, progenitors); catalase/glutathione PPARγ-independent	PPARγ-mediated; negligible CB_1_/CB_2_ affinity (K_i_ > 40 µM)	[69,122]
VCE-003.2	ALS	SOD1-G93A (mice)	Mouse (B6SJL-Tg(SOD1*G93A)1Gur/J, male, treated from 60 d; non-transgenic littermates); primary astrocytes (mouse) *n* = 4–6/group; ≥ 15 cells/condition (Sholl)	Preserved spinal motor neurons; reduced astrogliosis; transient motor benefit; normalized IL-1β and CB_2_	Not performed (inferred)	PPARγ-mediated (inferred; rosiglitazone parallelism only)	[70]
Δ^8^-THCV	SUD	Nicotine self-administration, CPP, relapses, withdrawal	Rat (alcohol-preferring P, male); mouse (ICR, male) *n* = 8–12/group (rat); 6–8/group (mouse)	Reduced nicotine self-administration, cue- and nicotine-induced relapse, CPP, and withdrawal signs; dose-related for self-administration and relapse	Not performed	CB_1_ antagonism/CB_2_ agonism consistent; not definitively proven	[123]

Antagonist/genetic validation: “validated”, blocking agent named; “partial”, target independence or receptor selectivity established but effector mechanism inferred; “not performed”. † Species inferred from the cited primary source rather than stated in the original model description. Sample sizes were small and not uniformly reported across studies; this limitation is discussed in Section 8. Δ^9^-THCA, by contrast, showed model-dependent results ranging from ineffective (Dravet hyperthermia, 6 Hz) to proconvulsant (MES) as a purified isolate, with anticonvulsant effects emerging only in combination with Δ^9^-THC [114]. Its further development is constrained by poor brain penetration and chemical instability during decarboxylation.

**Table 6:** Human and early-phase clinical evidence for minor phytocannabinoids with CNS relevance.

Compound	Indication	Design/ Sample	Dose/ Formulation	Primary Endpoint Result	Key Caveat(s)	Safety	Ref.
CBDV	Epilepsy (focal seizures)	Phase IIa randomized clinical trial, double-blind, placebo-controlled; *n* = 162 adults	800 mg oral twice daily (1600 mg·day^−1^); purified isolate (GWP42006)	Negative: seizure frequency reduced 40.5% vs. 38% placebo; no separation on primary or secondary endpoints	High placebo response; clinical dose 8–10-fold below effective preclinical dose (mg·kg^−1^); loss of potential entourage effects with purified isolate vs. botanical extract used preclinically	Generally well tolerated; treatment-emergent adverse events 72.8% vs. 48.1% (placebo); 2 discontinuations for transaminase elevation; no Hy’s Law cases	[135]
CBDV	ASD–neurochemistry (MRS)	Double-blind, placebo-controlled crossover; *n* = 34	Single 600 mg oral CBDV	Increased basal ganglia glutamate; change negatively correlated with baseline in ASD group	Single-dose; pharmacodynamic endpoint only; no symptomatic outcome assessed	Single-dose tolerability acceptable	[128]
CBDV	ASD–functional connectivity (fMRI)	Double-blind, placebo-controlled crossover; *n* = 28	Single 600 mg oral CBDV	Normalization of atypical striatal connectivity patterns	Surrogate endpoint; small sample; no behavioral measures	Acute tolerability acceptable	[128]
CBDV	Rett syndrome (pediatric safety)	Phase I tolerability; *n* = 5 girls	10 mg·kg^−1^·day^−1^ oral CBDV	Tolerability established; not powered for efficacy	Very small sample; short duration; pediatric safety only	Pediatric tolerability acceptable	[129]
CBDV	Neuropathic pain (HIV-associated)	Randomized, placebo-controlled; *n* = 32	400 mg·day^−1^ oral CBDV for 4 weeks	Directionally unfavorable: pain intensity numerically worse than placebo	Oral exposure likely insufficient; mechanism may not address neuropathic pathophysiology	No major safety signals reported	[126]
Δ^9^-THCV	Attenuation of Δ^9^-THC effects	Placebo-controlled crossover; *n* = 10 healthy male cannabis users	10 mg·day^−1^ oral for 5 days + 1 mg intravenous Δ^9^-THC challenge	Reduced subjective intoxication (9/10 subjects) and heart rate; memory intrusions increased; CAPE unchanged	Underpowered (*n* = 10); conservative dosing; mixed cognitive profile (verbal recall improved but intrusions increased)	No serious adverse events reported	[135]
Δ^9^-THCV	Neural reward processing	Single-dose fMRI; *n* = 20 healthy volunteers	Single 10 mg oral Δ^9^-THCV	Increased activation to rewarding and aversive stimuli in midbrain, cingulate, and striatum	Single-dose; surrogate neuroimaging endpoint; no clinical outcome	Acute tolerability acceptable	[136]
Δ^9^-THCV	Type 2 diabetes (metabolic endpoints)	Randomized, double-blind, placebo-controlled parallel-group pilot; *n* = 62	10 mg·day^−1^ Δ^9^-THCV for 13 weeks (monotherapy and CBD combination arms)	Primary high-density lipoprotein endpoint not met; Δ^9^-THCV monotherapy improved several secondary glycemic endpoints; CBD co-administration abolished these benefits	Pilot size; primary endpoint negative; CBD–Δ^9^-THCV antagonism demonstrates a pharmacokinetic or pharmacodynamic interaction	No serious adverse events over 13 weeks; no weight change	[53]
Δ^8^-THCV	Phase I safety (healthy volunteers)	Phase I, double-blind, placebo-controlled, dose-ranging	12.5–50 mg oral	No efficacy endpoints; tolerability across dose range	Safety data only; no CNS efficacy assessment	No serious adverse events across dose range	[132]
CBN	Sleep/sedation	Human volunteer study; *n* = 5 males	50 mg oral CBN ± Δ^9^-THC	CBN alone inactive on all measures; combination modestly increased subjective drowsiness vs. Δ^9^-THC alone	Very small sample; outdated methodology (1975); no polysomnography; no placebo-only arm	No major adverse events reported	[137]
CBN	Sleep quality	Randomized, double-blind, placebo- and melatonin-controlled, decentralized; *n* = 1020 adults (202–206 per arm)	25–100 mg oral purified CBN (three formulations)	Reduced self-reported sleep disturbance vs. placebo (all three doses); no separation from 4 mg melatonin	Self-reported endpoint; no polysomnographic confirmation; no separation from placebo in the proportion achieving the minimal clinically important difference for any arm, including melatonin; sponsor-conducted and industry-funded; decentralized design	Tolerability comparable to melatonin	[138]
CBG	Anxiety and stress (acute)	Double-blind, placebo-controlled crossover field trial; *n* = 34 healthy adults	Single 20 mg oral CBG (hemp-derived tincture)	Reduced acute anxiety (20, 45, and 60 min post-dose) and stress vs. placebo; enhanced verbal memory; no subjective drug effects	Single acute dose; remote field-trial design; cannabis-using sample; no clinical anxiety-disorder population	No intoxication or impairment; no serious adverse events reported	[133]

All the studies listed are early-phase. Except for the CBDV epilepsy trial, none were powered for efficacy, and several reported only surrogate or pharmacodynamic endpoints. GWP42006, purified CBDV investigational product; Hy’s Law, regulatory criterion for potential drug-induced liver injury.

**Table 7:** Proposed biomarker development roadmap for minor cannabinoid clinical translation: compound-specific modalities, validation status, and recommended integration stage.

Compound– Indication	Proposed Biomarker	Modality	Validation Status and Precedent	Recommended Stage	Key Methodological Consideration
CBDV–ASD	Basal ganglia glutamate normalization	^1^H-MRS	Demonstrated in autistic adults with CBDV [128]; requires correlation with symptomatic endpoints	Phase II proof-of-concept	Glutamate quantification sensitive to voxel placement and field strength; multi-site harmonization needed
CBDV–ASD	Striatal functional connectivity shift toward neurotypical profile	resting-state functional MRI	Demonstrated in autistic adults with CBDV [129]; surrogate endpoint status unestablished	Phase II proof-of-concept	Correlation with validated behavioral outcome measures (e.g., Social Responsiveness Scale-2, Aberrant Behavior Checklist) required for regulatory acceptance
CBDV–Epilepsy	CSF or plasma CBDV exposure confirmation	Pharmacokinetic sampling	Not yet performed; addresses the dose–exposure gap identified as primary translational barrier (Section 7.4)	Phase I/II dose-finding	Would determine whether clinical doses achieve CNS concentrations comparable to effective preclinical exposures
Δ^9^-THCV–Psychosis	CB_1_ receptor occupancy	PET ([^18^F]MK-9470 or [^18^F]FMPEP-d_2_)	[^18^F]MK-9470: IC_50_ 0.7 nM, 7% test–retest variability in humans [155]; [^18^F]FMPEP-d_2_: superior precision, smaller sample sizes [155]	Phase I dose-finding	THCV is a putative neutral (vs inverse) antagonist; validated radioligands are inverse agonists, so competition kinetics may differ from standard agonist-displacement paradigms
Δ^9^-THCV–Psychosis	Reward/aversion circuit activation	Task fMRI	Feasibility established in healthy volunteers with THCV [137]	Phase II proof-of-concept	Extend to clinical psychosis cohort; test whether CB_1_ antagonism normalizes aberrant salience signaling
Δ^8^-THCV–Nicotine addiction	Cue-induced craving and reward-circuit reactivity	Cue-reactivity fMRI	Standard paradigm in addiction neuroscience; not yet applied to Δ^8^-THCV	Phase II proof-of-concept	Correlate with self-reported craving and smoking reduction endpoints
CBDA-ME–Anxiety	Amygdala reactivity to threat under stress priming	Task fMRI	Standard paradigm in anxiety pharmacology [156]; not yet applied to cannabinoids	Phase II proof-of-concept	Stress-priming protocol essential to test the state-dependent efficacy demonstrated preclinically [87]
VCE-003.2–Neurodegeneration	CSF neuroinflammatory cytokine panel (primary analytes: IL-1β, TNF-α)	Immunoassay	Preclinical relevance established [70,121]; translatable to human lumbar CSF sampling	Phase I/II safety-efficacy	Baseline and post-treatment paired sampling; correlate with clinical progression markers
VCE-003.2–Neurodegeneration	Microglial activation	TSPO PET (e.g., [^18^F]DPA-714)	Established neuroinflammation imaging marker; second-generation tracers reduce non-specific binding	Phase II proof-of-concept	rs6971 TSPO polymorphism affects [^18^F]DPA-714 binding; genotype-stratified analysis required. Third-generation polymorphism-insensitive tracers (e.g., [^18^F]BS224) may simplify future study designs.
CBDV/Δ^9^-THCV–Multiple indications	CYP2D6/CYP2C19 genotype	Pharmacogenomic testing	Ref. [153] demonstrated significant binding affinity variation across four CYP2D6 polymorphic variants for CBDV and THCV	Phase I/II dose-finding	Incorporate into dose-finding protocols to reduce pharmacokinetic variability; particularly important given the narrow therapeutic window implied by CBDV trial data (Section 7.5)

## Data Availability

No new data were created or analyzed in this study. Data sharing is not applicable to this article.

## Abbreviations

The following abbreviations are used in this manuscript:

2-AG	2-Arachidonoylglycerol
3-NP	3-Nitropropionic acid
5-HT_1_A	5-Hydroxytryptamine receptor type 1A
6-OHDA	6-Hydroxydopamine
^1^H-MRS	Proton magnetic resonance spectroscopy
AEA	Anandamide
ALS	Amyotrophic lateral sclerosis
ASD	Autism spectrum disorder
CBC	Cannabichromene
CBD	Cannabidiol
CBDA	Cannabidiolic acid
CBDA-ME	Cannabidiolic acid methyl ester
CBDV	Cannabidivarin
CBG	Cannabigerol
CBGA	Cannabigerolic acid
CBN	Cannabinol
CB_1_	Cannabinoid receptor type 1
CB_2_	Cannabinoid receptor type 2
CNS	Central nervous system
COX-2	Cyclooxygenase-2
CPP	Conditioned place preference
CSF	Cerebrospinal fluid
CYP	Cytochrome P450
EAE	Experimental autoimmune encephalomyelitis
EC_50_	Half-maximal effective concentration
ERK	Extracellular signal-regulated kinase
FAAH	Fatty acid amide hydrolase
FDA	U.S. Food and Drug Administration
fMRI	Functional magnetic resonance imaging
GABA_A_	γ-aminobutyric acid type A receptor
GIRK	G-protein-gated inwardly rectifying potassium channel
GPCR	G-protein-coupled receptor
GPR55	G-protein-coupled receptor 55
HD	Huntington’s disease
HIV	Human immunodeficiency virus
IC_50_	Half-maximal inhibitory concentration
IL-1β	Interleukin-1β
IL-6	Interleukin-6
IL-8	Interleukin-8
IND	Investigational new drug
iNOS	Inducible nitric oxide synthase
Kᵢ	Inhibition constant
LPS	Lipopolysaccharide
MAGL	Monoacylglycerol lipase
MES	Maximal electroshock
MRS	Magnetic resonance spectroscopy
MS	Multiple sclerosis
Na_V_	Voltage-gated sodium channel
Neurodev.	Neurodevelopmental disorders
NF-κB	Nuclear factor κB
NREM	Non-rapid eye movement
Nrf2	Nuclear factor erythroid 2–related factor 2
PAM	Positive allosteric modulator
PD	Parkinson’s disease
PET	Positron emission tomography
PPARγ	Peroxisome proliferator-activated receptor gamma
PRIME	Priority Medicines scheme
PRISMA	Preferred Reporting Items for Systematic Reviews and Meta-Analyses
PTZ	Pentylenetetrazole
SUD	Substance use disorder
TMEV	Theiler’s murine encephalomyelitis virus
TNF-α	Tumor necrosis factor-α
TRP	Transient receptor potential
TRPA1	Transient receptor potential ankyrin 1
TRPM8	Transient receptor potential melastatin 8
TRPV1	Transient receptor potential vanilloid 1
TRPV2	Transient receptor potential vanilloid 2
TRPV4	Transient receptor potential vanilloid 4
TSPO	Translocator protein
α_2_-AR	α_2_-Adrenergic receptor
Δ^8^-THCV	Δ^8^-Tetrahydrocannabivarin
Δ^9^-THC	Δ^9^-Tetrahydrocannabinol
Δ^9^-THCA	Δ^9^-Tetrahydrocannabinolic acid
Δ^9^-THCV	Δ^9^-Tetrahydrocannabivarin
